# In Situ Scanning Electron Microscopy Crack Characterization and Resistance Evolution in Cyclically-Strained Ag Nanoflake-Based Inks

**DOI:** 10.1021/acsanm.4c05133

**Published:** 2024-11-25

**Authors:** Qiushi Li, Antonia Antoniou, Olivier N. Pierron

**Affiliations:** G.W. Woodruff School of Mechanical Engineering, Georgia Institute of Technology, Atlanta, Georgia 30318, United States

**Keywords:** nanocomposites, conductors, fatigue, cracking, strain maps, flexible electronics

## Abstract

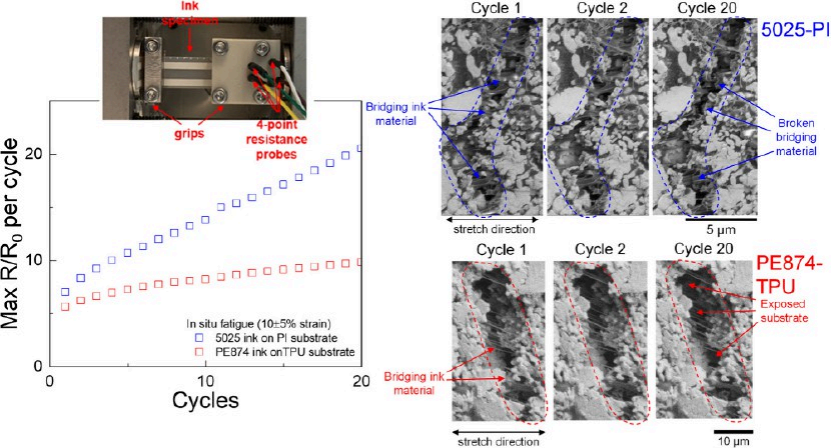

The reliability of nanocomposite conductive inks under cyclic loading is the key to designing robust flexible electronics. Although resistance increases with cycling and models exist, the exact degradation mechanism is not well understood and is critical for developing inks. This study links cracking behavior to changes in electrical resistance by performing in situ cyclic stretch experiments in scanning electron microscopy (SEM) with synchronized resistance measurements. Two screen-printed conductive inks, PE874 and 5025, on thermoplastic polyurethane (TPU) and polyimide (PI) substrates, respectively, were tested using the in situ technique. The obtained SEM images were analyzed with digital image correlation (DIC) to map the strain across cycles. The strain maps show that fatigue damage mainly occurred within the cracks formed during the initial monotonic stretch. There was no delamination at the ink–substrate interface or crack extension along the surface with cycling. Instead, fatigue damage resulted from a combination of crack widening and local shearing within the existing cracks. Crack depth varied based on the ink and substrate properties. The cracks in the 5025 ink on the PI substrate were only partially through the ink thickness, while fully through-thickness cracks were more prevalent in the PE874 ink on the TPU substrate. The 5025 ink showed a faster resistance increase with cycling than the PE874 ink because fatigue damage affected more bridging ink material for partial through-thickness cracks. Higher strain amplitudes caused greater crack widening and shearing and therefore faster resistance increase per cycle.

## 1 Introduction

Flexible hybrid electronics (FHE) devices integrate electronic components with a compliant circuity conformable to nonplanar surfaces. Electrical signals are transmitted across the circuitry via conductive interconnects printed on a flexible or stretchable polymer substrate. The conformable and lightweight characteristics of FHE device designs have enabled their notable applications as wearable healthcare devices,^1−5^ which can monitor healthcare data such as the vital signs or muscular activity without limiting the movement of the wearer. These devices are expected to undergo repeated deformation during the wearer’s daily activities, which could include stretching, bending, and twisting deformation. For stretching deformation, repeated straining of up to 30–50%^6^ could be expected during typical use. The successful functioning of flexible electronics devices requires interconnecting materials to maintain conductivity under such repeated deformation. Therefore, it is imperative to gain a detailed understanding of the electromechanical behavior for these materials, especially under fatigue loading conditions.

Several different classes of materials have been studied for their electromechanical behavior as electrical interconnects in flexible electronics devices, including thin metallic films,^7−18^ printed nanoparticles,^19−22^ and metal–polymer composites.^23−38^ Thin metallic films deposited on polymer substrates have high initial electrical conductivity but suffer rupture or debonding at moderate strain levels (∼10%) under tensile stretching due to the film–substrate mismatch in elastic properties. In comparison, metal–polymer composites have much lower stiffness than homogeneous metallic thin films and larger ductility and, therefore, can be stretched to higher strains without rupture.^5,23,27,31,35−38^ One common type of metal–polymer composite, also known as nanocomposite conductive inks, consists of nm- to μm-sized metal flakes surrounded by a polymer binder material. The conductive ink is then printed as a film (with a thickness of ∼10 μm) onto a thicker polymer substrate, usually by screen printing. The two conductive inks studied in the current work are PE874 and 5025 inks, both formulated by DuPont. The PE874 ink consists of Ag flakes (with lateral sizes 100s of nm to 10s of μm and thickness around 50–100 nm)^23^ surrounded by a polyurethane binder material and has a significant volume of μm-sized pores (up to 17% of total ink volume).^23^ The 5025 ink consists of similar Ag flakes embedded in an acrylic binder material and has no porosity. The choice of the substrate materials for the two inks, which are typically the TE-11C thermoplastic polyurethane (TPU) for the PE874 ink and the Kapton polyimide (PI) for the 5025 ink, was made so that the stiffness of the substrate materials roughly matched those of the respective ink binder materials. The PE847 ink, which was typically printed on the compliant TPU substrate (with an initial elastic modulus of ∼20 MPa), was designed for stretchable applications. The 5025 ink, which was printed on the much stiffer PI substrate (with an initial elastic modulus of ∼2 GPa), was designed for flexible applications.

**Figure 1:**
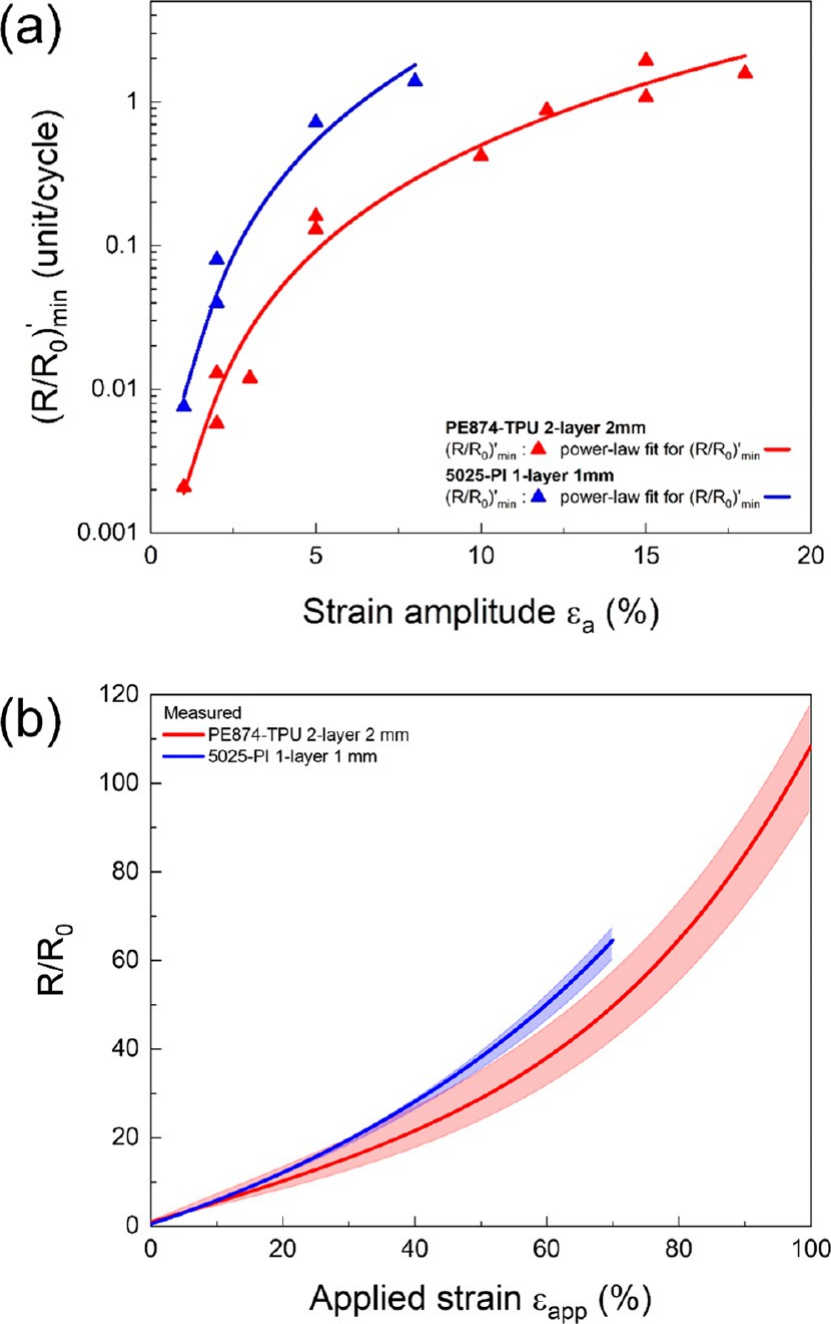
(a) (*R*/*R*_0_)_min_^′^ versus strain amplitude for cyclic stretch experiments with PE874 and 5025 inks. (b) Measured *R*/*R*_0_ evolution with applied strain for the two inks shown with standard deviation bounds.

Previous cyclic stretch experiments performed outside the scanning electron microscopy (SEM) (ex situ) showed different rates of *R*/*R*_0_ increase with cycling for the two inks.^39^ In these cyclic experiments, the *R*/*R*_0_ increase was characterized by a steady-state minimum rate of *R*/*R*_0_ increase with cycling or (*R*/*R*_0_)_min_^′^.^28,39^Figure 1(a) shows this minimum rate (*R*/*R*_0_)_min_^′^ for the two inks tested at various strain amplitudes.^39^ For the tested strain amplitudes, (*R*/*R*_0_)_min_^′^ was about 5 to 10 times higher for the 5025 ink compared to the PE874 ink. For both inks, (*R*/*R*_0_)_min_^′^ was found to have a strong correlation with the strain amplitude, while the mean strain had a much smaller effect on (*R*/*R*_0_)_min_^′^.^28,39,40^ The current work is motivated in part by the goal of understanding the material deformation mechanisms responsible for the different (*R*/*R*_0_)_min_^′^ rates observed in the ex situ experiments.

Strain localization has been known to play a key role in an increase in ink resistance with monotonic tensile strain. Previous uniaxial stretch experiments with the two inks have shown that the resistance increase with tensile strain far exceeded model predictions based on uniform ink material deformation. Figure 1(b) shows the *R*/*R*_0_ evolution with applied tensile strain ε_app_ for the two inks from experiments by Li et al.^39^ with standard deviation bounds based on at least three experiments for each ink. The measured *R*/*R*_0_ values for both inks far exceed the predicted *R*/*R*_0_ values that would result from strain-induced geometric dimensional changes alone. Experiments with in situ scanning electron microscope (SEM) imaging showed that the ink deformation was dominated by strain localization primarily in the form of cracking.^23,27,41^ Bands of strain localization were found to initiate at low ε_app_ (∼1%) when the ink is uniaxially stretched, evolving into visible surface cracks at higher ε_app_ (∼3% for PE874 and ∼5% for 5025 ink). The formation and growth of these cracks have a dominant impact on the electrical performance of the ink. Under cyclic uniaxial stretching, the cracks in the PE874 ink were shown in a previous in situ SEM experiment to undergo progressive widening with cycling but not extend further in-plane.^27^ Beyond these initial results, the relationship between the deformation mechanisms and the resistance increase with cyclic strain is still not well understood. The current work investigates the cracking mechanisms contributing to ink resistance during cyclic stretching with the help of in situ SEM cyclic stretch experiments.

The cracking mechanisms of the ink film can differ significantly due to the ink material properties. The PE874 ink, which has a low toughness polyurethane binder material and significant porosity, was shown by previous in situ monotonic stretch experiments to have cracks that quickly penetrated through the ink thickness with increasing ε_app_ (<15%).^41^ The 5025 ink, which has a tougher acrylic binder material and no porosity, was shown to have cracks that remained only partially through-thickness even at high ε_app_ (>30%).^41^ Though the cracks in the 5205 ink were only partially through the ink thickness, the cracks were longer, leading to larger measured *R*/*R*_0_ for the 5025 ink compared to the PE874 ink, especially at higher ε_app_ (>15%).^23,41^ At an ε_app_ of 30%, the measured normalized resistance *R*/*R*_0_ for the 5025 ink on the PI substrate was about 40% higher than that for the PE874 ink on the TPU substrate at the same ε_app_. Under cyclic stretching between two strain values, however, the difference in the *R*/*R*_0_ increases in the two inks was much more drastic. As shown in Figure 1(a), the *R*/*R*_0_ increase was about 200% to 600% higher for the 5025 ink than for the PE874 ink, depending on the strain amplitude used for stretching.^39^ The current work seeks to understand the deformation mechanisms responsible for the greater difference in electrical performance during cyclic stretching.

In addition, the substrate may have non-negligible impact on the ink cracking behavior, given that the stiff PI substrate provides more constraint to ink strain localization, thereby inhibiting crack penetration through the ink thickness.^29,41^ Under monotonic stretching, the measured *R*/*R*_0_ was close between the PE874 ink printed on the TPU substrate and that printed on the PI substrate up to an applied strain of 15%.^23^ However, the effect of the substrate on the cracking behavior and resistance increase in the case of cyclic stretching remains to be investigated. The current work examines the effect of the substrate on the cracking mechanisms and ink resistance increase with cyclic stretch for the PE874 ink. The in situ SEM experiments in the current work couple in situ SEM imaging with the synchronous measurement of electrical resistance during fatigue testing for the aforementioned conductive inks. First, the resistance increases for in situ SEM tests are confirmed to match the existing ex situ data^28,39^ in order to validate the use of the in situ SEM technique to investigate the fatigue mechanisms responsible for the resistance behavior. The in situ SEM images are analyzed quantitatively using digital image correlation to obtain relative increases in local strain at ink surface with cyclic stretching. The correspondence between cracking mechanisms and resistance increase provides valuable insight for designing conductive ink materials to minimize the resistance increase with cyclic deformation.

## 2 Experimental Section

### 2.1 Ink Properties and Specimen Geometry

**Figure 2:**
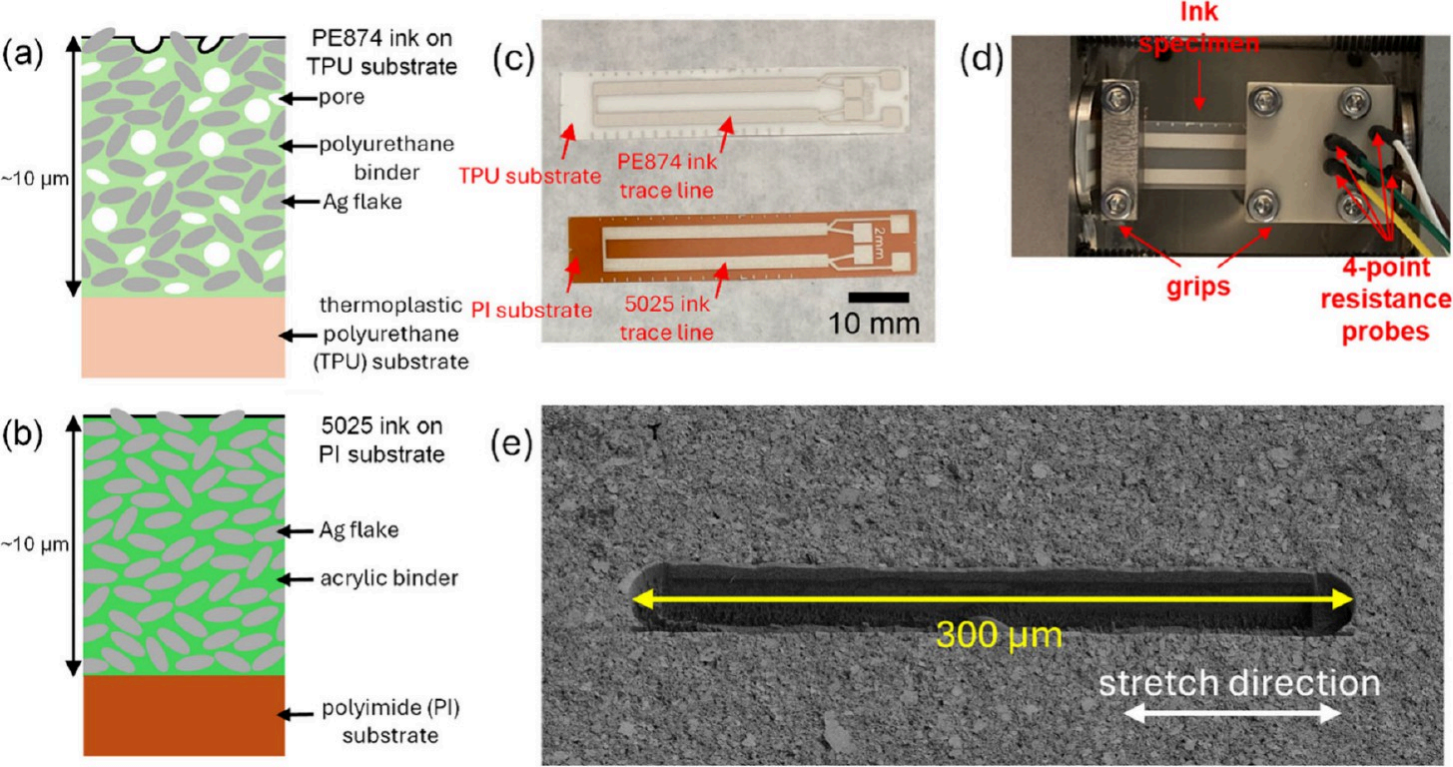
Schematics for the composite material structure of (a) PE874 ink deposited on thermoplastic polyurethane (TPU) substrate and (b) 5025 ink deposited on polyimide (PI) substrate; (c) PE874-TPU (top) and 5025-PI (bottom) specimens; (d) the electromechanical testing setup; (e) tilted view SEM image of FIB (focused ion beam) cut in PE874 ink for in situ testing.

The PE874 and 5025 conductive inks formulated by DuPont are used in the current work. The PE874 ink uses a polyurethane binder material, which has an elastic modulus of about 10 MPa, while the 5025 ink uses an acrylic binder material, which has a much higher elastic modulus of about 1 to 3 GPa. Both inks use the same type of Ag flakes, with flake sizes ranging from 100s of nm to 10s of μm. The total volume fraction occupied by the Ag flakes was higher for the PE874 ink at 55% than for the 5025 ink at 49%. The PE874 ink also has a high volume fraction (17%) of μm-sized pores, while the 5025 ink is not porous. The inks were screen-printed in a single printing pass onto their respective substrates, which are thermoplastic polyurethane (TPU) or polyimide (PI) for the PE874 ink and PI for the 5025 ink. The screen-printed inks were then cured at 130 °C for 15 min. For both inks, the ink film thickness was about 10 μm. The TPU substrate was about 89 μm thick, while the PI substrate was about 127 μm thick. For the ex situ and in situ cyclic stretch experiments, U-shaped double straight trace line specimens with pads for 4-point resistance measurement probes were used (Figure 2(c)). The specimens for both inks had a trace width of 2 mm, which was the widest available trace width and was found to have a negligible effect on crack propagation.^25,27^

### 2.2 In Situ SEM Experimental Protocol

The cyclic uniaxial stretch experiments were performed on the Kammrath & Weiss Mz0-1 tensile module at a strain rate of 0.15% per second. The U-shaped ink specimen is secured between the grips with an initial gauge length of 30 mm for the ex situ experiments and 15 mm for the in situ experiments (Figure 2(d)). While the ink specimen is stretched between the grips, the electrical resistance is measured synchronously via 4-point resistance measurement probes (Figure 2(b)). The in situ SEM experiments were performed with the tensile module placed inside the Thermo Fisher Scientific 5 CX FIB-SEM. During the initial monotonic stretching of the in situ SEM experiment, tensile strain ε_app_ is applied incrementally, with pauses for imaging at 500× for wide field (∼300 μm) images of the crack pattern and at higher magnification (2500 to 5000×) for close-up images of individual cracks. During the cyclic stretching of the PE874-TPU and 5025-PI specimen at 10 ± 5% strain, the experiments were paused at the maximum strain during cycles 1, 2, 3, 4, 5, 10, and 20 for the wide field and close-up imaging. During the cyclic stretching of the PE874-TPU specimen at 40 ± 15% strain, the specimen was cyclically stretched for 5 cycles, with the experiment being paused at the maximum strain during each cycle for the wide field and close-up imaging. A separate in situ cyclic stretch experiment was performed on a 1 mm wide PE874-TPU specimen with a FIB cross-section cut. A 300 μm-long FIB cross-section cut was made along the loading direction through the entire thickness of the ink layer prior to the experiment (Figure 2(e)). Cyclic stretching across the FIB cut section at 23 ± 13% strain was performed with the experiment being paused at the maximum strain during cycles 1, 2, 3, 4, 5, 10, 20, and 30 for imaging at 250× and 5000× at a 30° angle tilt.

### 2.3 In-Plane Strain Maps from SEM Images

The SEM images acquired from the in situ experiments are analyzed using the digital image correlation (DIC) method. The DIC analysis was performed using the Ncorr software^42^ on both the wide field and close-up images to obtain in-plane displacement and strain maps for the imaged ε_app_ levels. Unlike the DIC analysis of SEM images from in situ monotonic stretch experiments, the DIC analysis for the in situ cyclic experiments focused on the relative axial strain (Δε_*xx*_) and shear strain (Δε_*xy*_) between the image at maximum strain at cycle 1 and the images at the maximum strain during subsequent cycles. In-plane displacements and strain maps with a reference image at 0% applied strain were also used to assess the extent of the existing crack pattern at cycle 1 during subsequent cycling (Supporting Information, Figure S1).

## 3 Results and Discussion

### 3.1 Electrical Resistance Measurements and Rates of Resistance Increase

**Figure 3:**
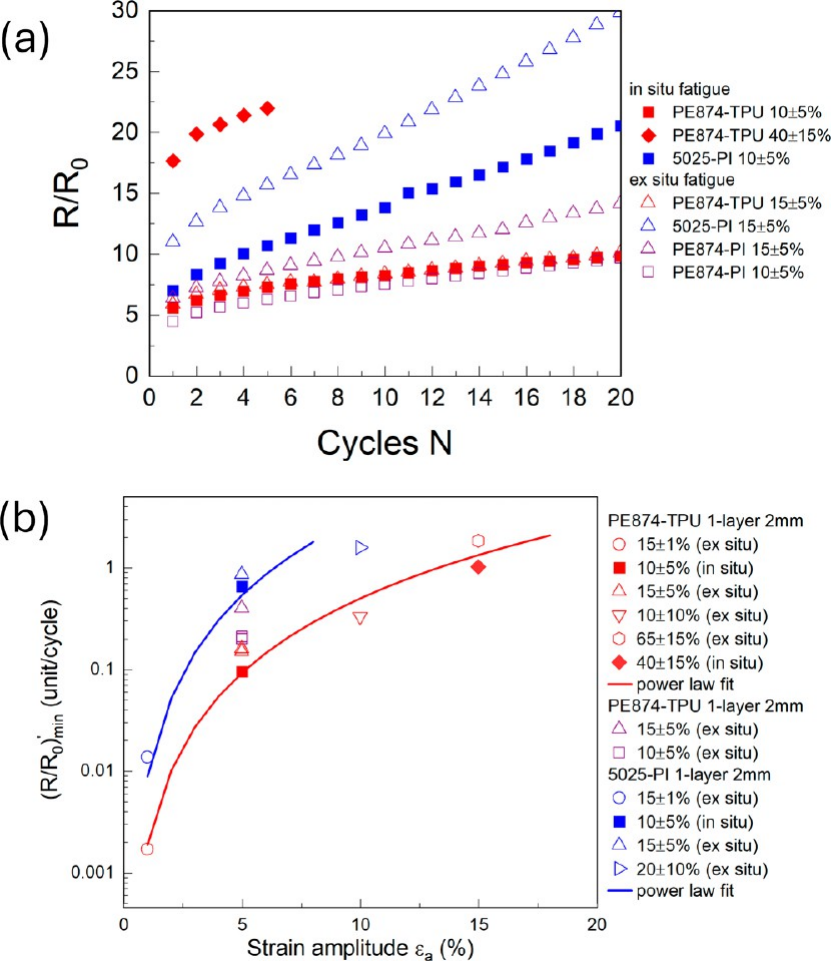
(a) Measured maximum *R*/*R*_0_ per cycle evolution over cycles for in situ SEM and ex situ cyclic stretch tests with 1-layer 2 mm trace width specimens, (b) (*R*/*R*_0_)_min_^′^ vs strain amplitude for in situ and ex situ tests with 1-layer 2 mm trace width specimens.

Figure 3(a) shows the evolution of the measured maximum *R*/*R*_0_ per cycle over the cycles for the three in situ SEM cyclic experiments considered in the current work, which include the PE874-TPU stretched at a mean strain of 10% and strain amplitude of 5% (10 ± 5%), the 5025-PI stretched at 10 ± 5% strain, and the PE874-TPU stretched at 40 ± 15% strain. The maximum *R*/*R*_0_ during cycle 1 for PE874-TPU and 5025-PI experiments at 10 ± 5% strain were similar at 5.58 and 7.01, respectively, as expected for the initial monotonic stretch.^23,41^ However, the rate of *R*/*R*_0_ increase with cycling (*R*/*R*_0_)′ was much higher for the 5025-PI than for the PE874-TPU for the same strain amplitude of 5%. When cycled at 10 ± 5% strain, the minimum rate (*R*/*R*_0_)_min_^′^ during the first 20 cycles was 0.65 for the 5025-PI in situ test compared to 0.095 for the PE874-TPU in situ test. A further ex situ test with the PE874-PI cycled at 10 ± 5% strain showed that, although *R*/*R*_0_ at maximum strain during the initial cycle was slightly lower for PE874-PI (4.4 compared to 5.6 for the PE874-TPU), (*R*/*R*_0_)_min_^′^ was twice that for the PE874-TPU in situ experiment (0.20 compared to 0.095 for the PE874-TPU). (*R*/*R*_0_)_min_^′^ during the first 5 cycles of the PE874-TPU stretched at 40 ± 15% strain was 1.02, which is about 10 times (*R*/*R*_0_)_min_^′^ for the PE874-TPU stretched at 10 ± 5% strain. The measured results for the ex situ experiments at 15 ± 5% strain are also shown for PE874-TPU, 5025-PI, and PE874-PI, which enable the comparison of the three ink–substrate combinations under the same ex situ testing conditions. The general trend in *R*/*R*_0_ magnitude for 5025-PI, PE874-PI, and PE874-TPU in descending order is also demonstrated for the ex situ tests at 15 ± 5% strain. Figure 3(b) shows (*R*/*R*_0_)_min_^′^ for various ex situ and in situ experiments at different strain amplitudes. The power law fits obtained by Li et al.^39^ (Figure 1(a)) for PE874-TPU and 5025-PI are superposed on Figure 3(b) and show good fits with the current results using one-layer 2 mm specimens, indicating that the difference in (*R*/*R*_0_)′ due to the geometric dimensions (2-layer versus 1-layer for the PE874-TPU and 1 mm versus 2 mm trace width for the 5025-PI) is negligible. The (*R*/*R*_0_)_min_^′^ values show a strong correlation with strain amplitude for both inks, while the effect of the mean strain on (*R*/*R*_0_)_min_^′^ is relatively minor. The (*R*/*R*_0_)_min_^′^ values from the in situ experiments showed a close match with those from the ex situ experiments at the same strain amplitude, thereby validating the in situ SEM technique to further explore the difference in fatigue degradation mechanisms leading to these significant differences in rates between (1) the PE874 ink on TPU substrate at strain amplitudes of 5% versus 15% and (2) the PE874 ink on TPU substrate versus the 5025 ink on PI substrate at a strain amplitude of 5%. For the 15 ± 5% strain tests, (*R*/*R*_0_)_min_^′^ for the PE874-PI test (0.40) was lower than that for the 5052-PI test (0.86) but higher than that for the PE874-TPU test (0.15). Hence, (*R*/*R*_0_)_min_^′^ for PE874-PI was intermediate between those of 5025-PI and PE874-TPU, demonstrating that both the ink and substrate materials affect the *R*/*R*_0_ increase with cycling.

### 3.2 In-Plane Strain Maps Using Low Magnification Images: Crack Pattern Evolution

**Figure 4:**
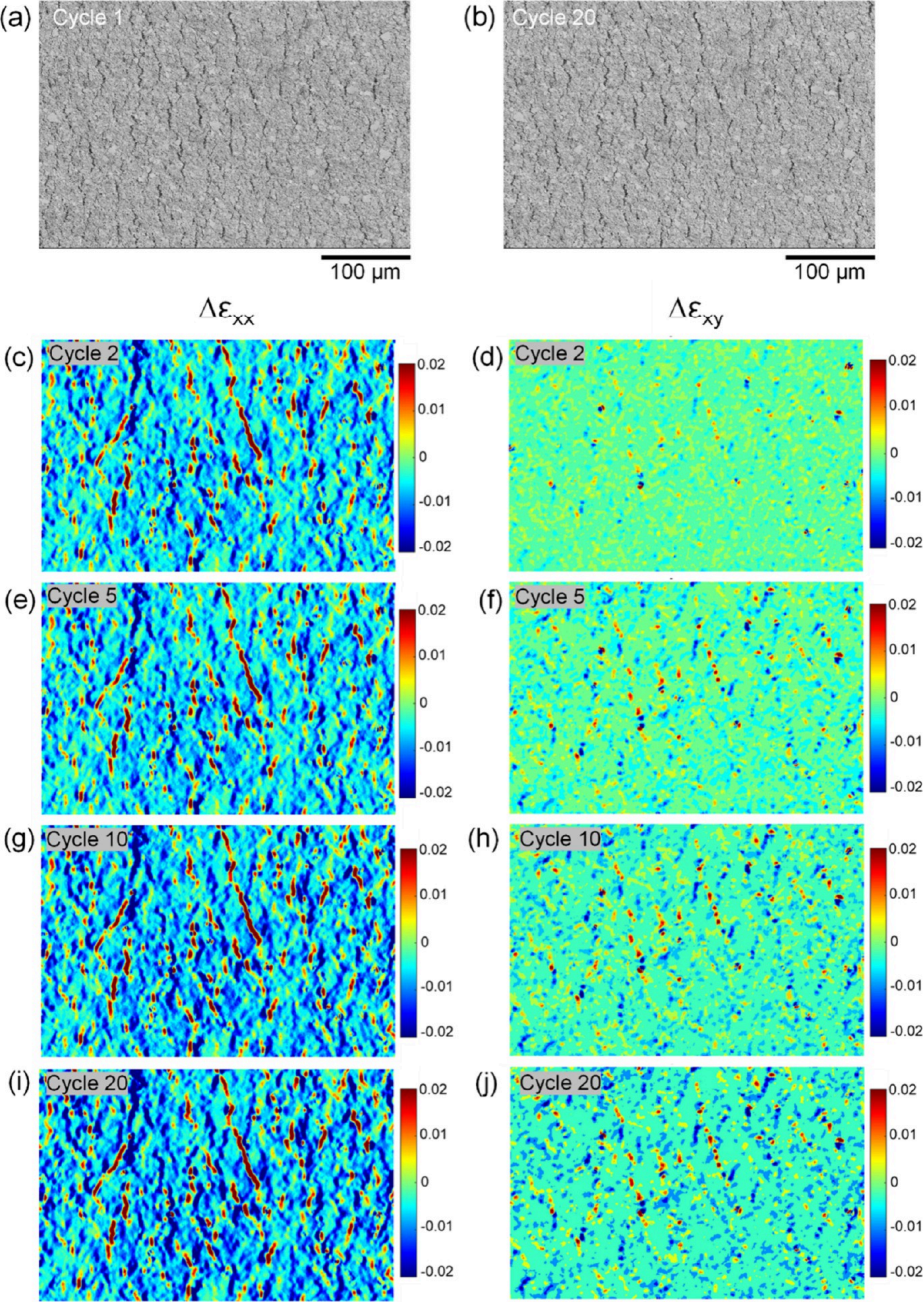
For PE874-TPU in situ SEM fatigue test at 10 ± 5% strain: 500× images at maximum strain during (a) cycle 1 and (b) cycle 20; relative tensile strain Δε_*xx*_ maps for (c) cycle 2, (e) cycle 5, (g) cycle 10, and (i) cycle 20; relative shear strain Δε_*xy*_ maps for (d) cycle 2, (f) cycle 5, (h) cycle 10, and (j) cycle 20 (same length scale as the 500× images).

**Figure 5:**
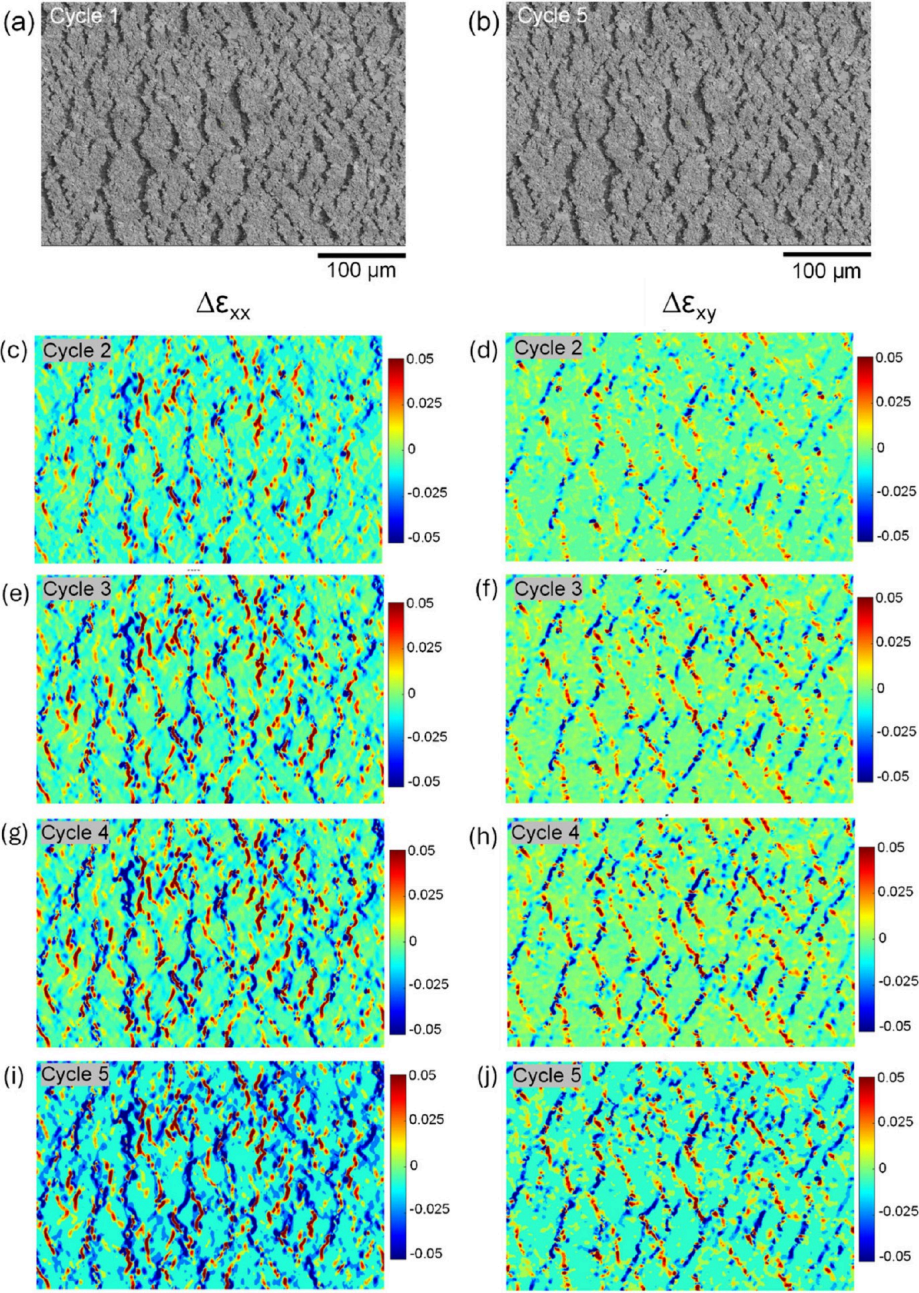
For PE874-TPU in situ SEM fatigue test at 40 ± 15% strain: 500× images at maximum strain during (a) cycle 1 and (b) cycle 20; relative tensile strain Δε_*xx*_ maps for (c) cycle 2, (e) cycle 5, (g) cycle 10, and (i) cycle 20; relative shear strain Δε_*xy*_ maps for (d) cycle 2, (f) cycle 5, (h) cycle 10, and (j) cycle 20 (same length scale as the 500× images).

Figure 4 shows, for the in situ cyclic stretch experiments for PE874-TPU at 10 ± 5% strain, respectively, the 500× images taken at maximum strain during cycles 1 and 20 (Figure 4(a,b)) and the relative strain maps over the cycles for the relative axial strain Δε_*xx*_ and relative shear strain Δε_*xy*_ (Figure 4(c–j)). Figures 5 shows, for a PE874-TPU experiment at 40 ± 15% strain, the analogous set of 500× images (Figure 5(a,b)) and relative strain maps (Figure 5(c–j)) for Δε_*xx*_ and Δε_*xy*_. For both the 10 ± 5% and 40 ± 15% strain tests, the relative strain maps show that the magnitude of Δε_*xx*_ and Δε_*xy*_ increased steadily for many cracks over the cycles. The values of the relative axial strain Δε_*xx*_ increased for some of the cracks that had positive Δε_*xx*_ but decreased for other cracks that had negative Δε_*xx*_, showing that some cracks were widening, while other cracks were narrowing. The number of widening and narrowing cracks is about the same (Supporting Information Figures S2, S3, and S4), suggesting a redistribution of relative crack opening widths with cyclic loading. Therefore, fatigue damage to the intact ink material accumulates by mixed mode I (crack widening) and II (crack shearing) crack displacements. The mixed mode fatigue damage causes a rearrangement of the crack openings with cycling, causing widening of some cracks and narrowing of others.

**Figure 6:**
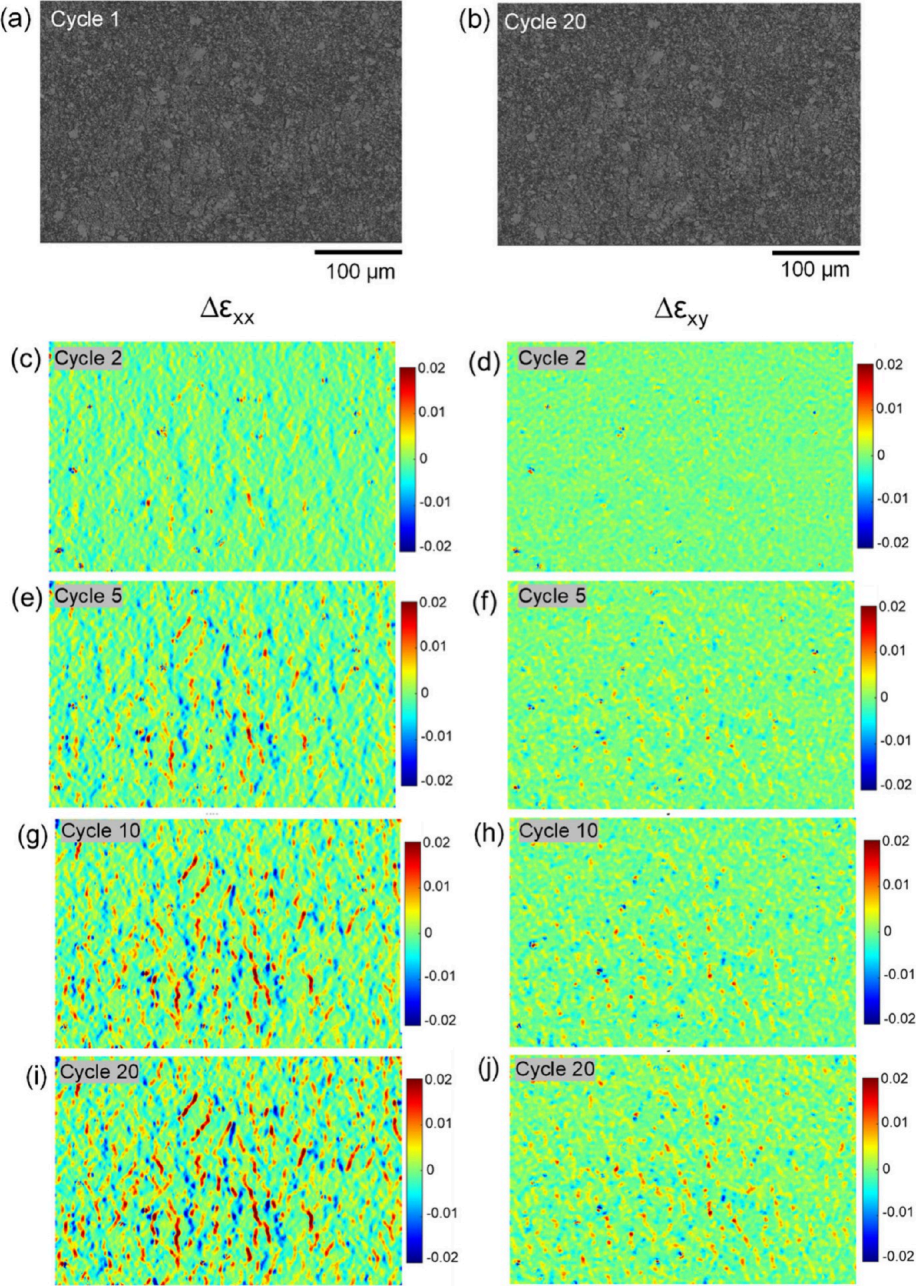
For 5025-PI in situ SEM fatigue test at 10 ± 5% strain: 500× images at maximum strain during (a) cycle 1 and (b) cycle 20; relative tensile strain Δε_*xx*_ maps for (c) cycle 2, (e) cycle 5, (g) cycle 10, and (i) cycle 20; relative shear strain Δε_*xy*_ maps for (d) cycle 2, (f) cycle 5, (h) cycle 10, and (j) cycle 20 (same length scale as the 500× images).

The regions of high relative strain magnitude correspond to the cracks undergoing widening, narrowing, or shear displacements. The maximum magnitude of local relative strain (peak values in Supporting Information Figures S2(k,l) and S3(k,l)) at cycle 5 for the 40 ± 15% test was about Δε_*xx*_ ∼ 0.5 and Δε_*xy*_ ∼ 0.2, which are about 10 times the relative strain peaks at cycle 5 for the 10 ± 5% test. Correspondingly, the (*R*/*R*_0_)′ value of 1.1 over the first 5 cycles for the 40 ± 15% test is about 10 times the (*R*/*R*_0_)′ over the first 5 cycles of the 10 ± 5% test, which is 0.13. Therefore, for the PE874 ink on the TPU substrate, a direct correlation appears to be present between the magnitude of the relative axial and shear strain on one hand and the rate of electrical resistance increase on the other. Figure 6 shows for a 5025-PI experiment at 10 ± 5% strain the analogous set of 500× images (Figure 6(a,b)) and relative strain maps (Figure 6(c–j)) for Δε_*xx*_ and Δε_*xy*_. The magnitude of Δε_*xx*_ and Δε_*xy*_ for the cracks in the 5025 ink showed a similar trend of increasing over the cycles as those in the PE874 ink. The relative axial strain Δε_*xx*_ also increased for cracks that widened and decreased for cracks that narrowed.

Under monotonic stretching, the resistance increase was closely related to the in-plane extension of the cracks with the applied strain. In the case of cyclic stretching, fatigue damage contributing to the resistance increase occurs without visibly extending the crack pattern established during the initial monotonic stretch (Supporting Information Figure S1). According to a model for *R*/*R*_0_ based on the crack pattern,^41^ the increases in *R*/*R*_0_ due to in-plane crack extension over the cycles was only up to about 2 for the 5025 ink and even less for the PE874 ink, which were much smaller compared to the increases in measured *R*/*R*_0_ over the cycles. The increase in ink resistance comes from the fatigue damage to the intact ink material (in the form of bridging ink material) within the existing crack pattern, causing the separation of once connected Ag flakes by the widening and shearing of the cracks.

### 3.3 In Situ Close-Up Imaging of Cracks

**Figure 7:**
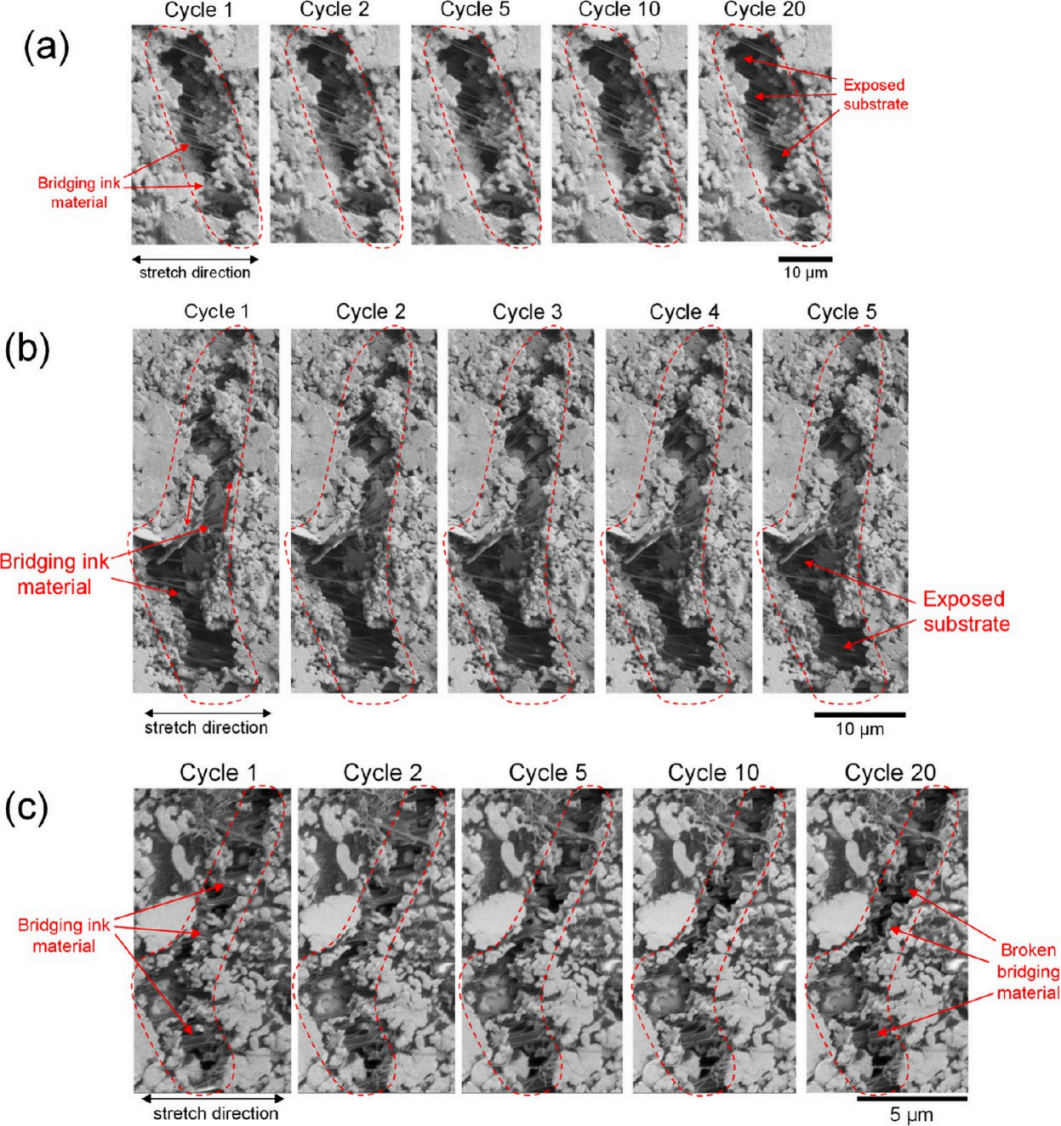
Close-up images over cycles for representative widening cracks from (a) PE874-TPU test at 10 ± 5% strain, (b) PE874-TPU test at 40 ± 15% strain, and (c) 5025-PI test at 10 ± 5% strain.

Figure 7 shows close-up images of representative individual cracks at maximum strain over the cycles from experiments of the PE874-TPU cycled at 10 ± 5% and 40 ± 15% strain and the 5025-PI cycled at 10 ± 5% strain. The region between the crack faces, which may have bridging ink material or otherwise be completely open, showing the exposed substrate, are marked by the dashed line enclosures. All of the cracks in the close-up images showed progressive widening and an increase in Δε_*xx*_ with cyclic stretching. For the P874-TPU experiments, though the cracks shown in Figure 7(a,b) had comparable crack widths, the cracks in Figure 7(b) had higher Δε_*xx*_ due to the higher strain amplitude (see strain maps in Supporting Information Figures S5 and S6). The cracking behavior of the PE874 ink on the TPU substrate is significantly different from that of the 5025 ink on the PI substrate. At the maximum strain of 15%, large portions of cracks in the PE874 ink have visibly penetrated through the ink thickness, exposing the substrate, like that shown in Figure 7(a). At the same strain level, all of the cracks in the 5025 ink are only partially through the ink thickness and have significant conductive bridging ink material between the crack faces, such as that shown in Figure 7(c). Upon cyclic stretching, the cracks in both inks widened, but more damage to the conductive ink material was done in the case of the 5025 ink. This is due to the larger volume of bridging ink material between the crack faces, which becomes progressively damaged with cyclic straining (Figure 7(c)). A second mode of fatigue damage occurs by shearing of the crack faces. The crack in Figure 7(b) also showed simultaneous widening and shearing along the crack faces, as marked by the set of arrows in opposite directions (see also Supporting Information Figure S7). Generally, more shearing damage should be expected for higher strain levels, at which the crack orientations become more slanted (Figures 4 and 5(a,b)).

### 3.4 Images and Strain Maps of Ink Cross-Section under Cyclic Strain

**Figure 8:**
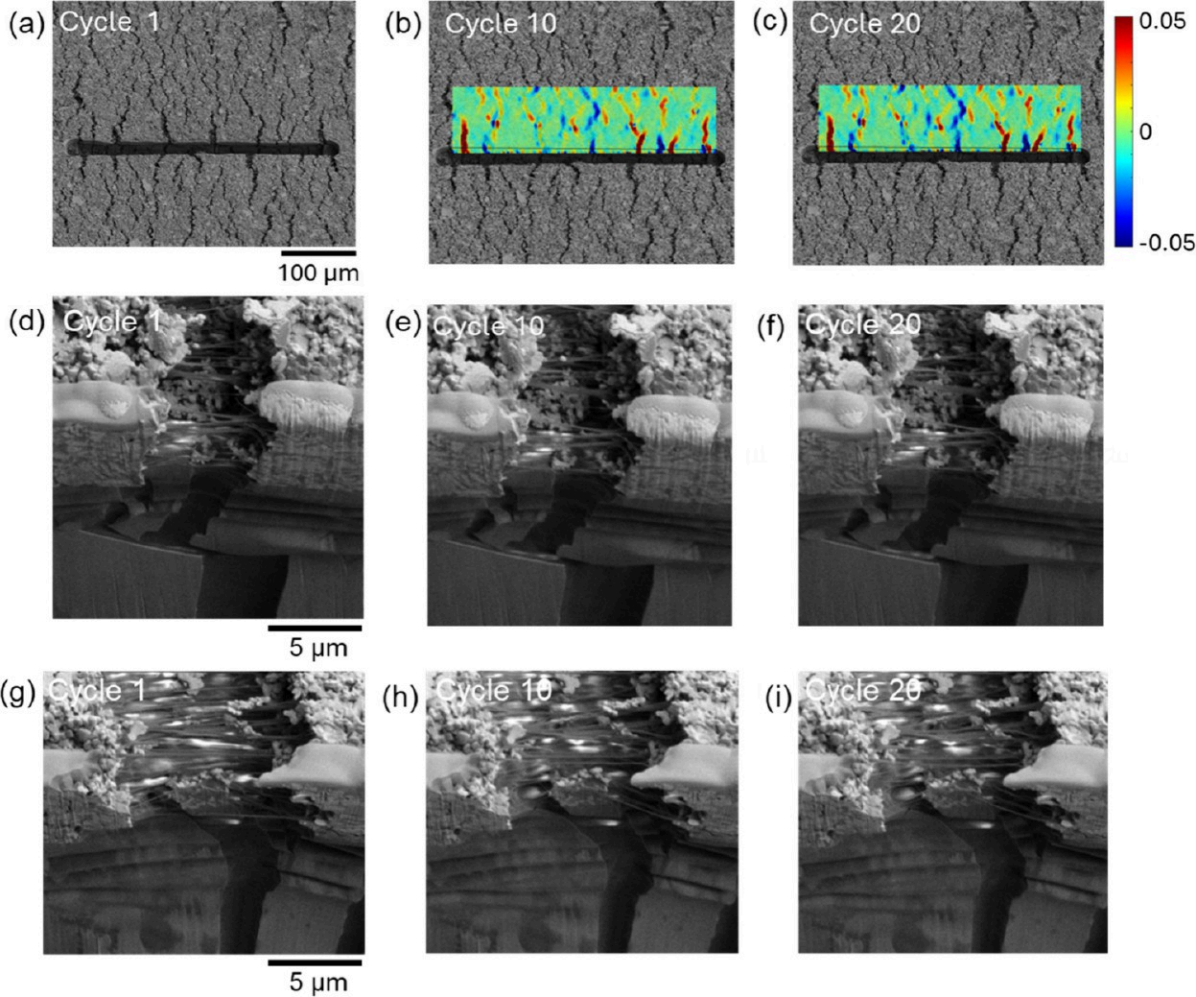
For the PE874-TPU in situ fatigue test at 23 ± 12% strain with FIB cut: (a) low magnification image at maximum strain during cycle 1; (b, c) low magnification images overlaid with relative tensile strain maps over the cycles; (d–f) close-up images of widening crack over the cycles; (g–i) close-up images of narrowing crack over the cycles.

Figure 8 shows the results from the in situ SEM experiment with the 1 mm wide PE874-TPU specimen cyclically stretched at 23 ± 12% strain across the 300-μm long FIB cross-section cut, which was made into the ink prior to the experiment. The strain maps, which have the 350× images (tilted at 30°) at maximum strain during cycle 1 as the reference image and those at maximum strain during subsequent cycles as current images, show the widening of some cracks and the narrowing of cracks at the FIB cross-section, as was observed in other in situ cyclic experiments. The cracks that widened with cycling were generally narrow initially, while the cracks that narrowed with cycling were initially wide, signifying a progressive adjustment of the initial crack configurations with cyclic straining. It is also worth noting that even at the high strain level of 35% there was no delamination between the ink film and the substrate. This is significant since the tensile stretching exerts a transverse load along the top part of the FIB cut that pushes the ink film in the transverse direction, but the ink film did not delaminate under the transverse load. Therefore, it can be inferred that delamination does not play a role in ink fatigue damage even at high applied strain levels (∼35%).

### 3.5 Discussion: Effect of Ink and Substrate Material Properties

The (*R*/*R*_0_)_min_^′^ results for the different ink–substrate combinations (Figure 3) show that both the ink and substrate material properties can have a significant impact on the ink cracking mechanisms during cyclic stretching, which lead to the different measured ink resistance evolutions for the two inks. The PE874 ink consists of Ag flakes surrounded by a relatively low volume of polyurethane binder (about 28% of the total volume)^23^ and a significant volume of μm-sized pores (about 17% of the total volume), while the 5025 ink has a high volume of acrylic binder (more than 50% of total volume) and has no porosity. As a result, PE874 ink is less resistant to crack propagation than the 5025 ink, and through-thickness cracking is more prevalent in the PE874 ink compared to the 5025 ink, which has only partial through-thickness cracks even at high applied strains.^41^ Upon cyclic straining, there was more bridging conductive material in the 5025 ink that could be damaged by the progressive widening and shearing of cracks, leading to the higher rate of *R*/*R*_0_ increase with cycling found for the 5025 ink.

**Figure 9:**
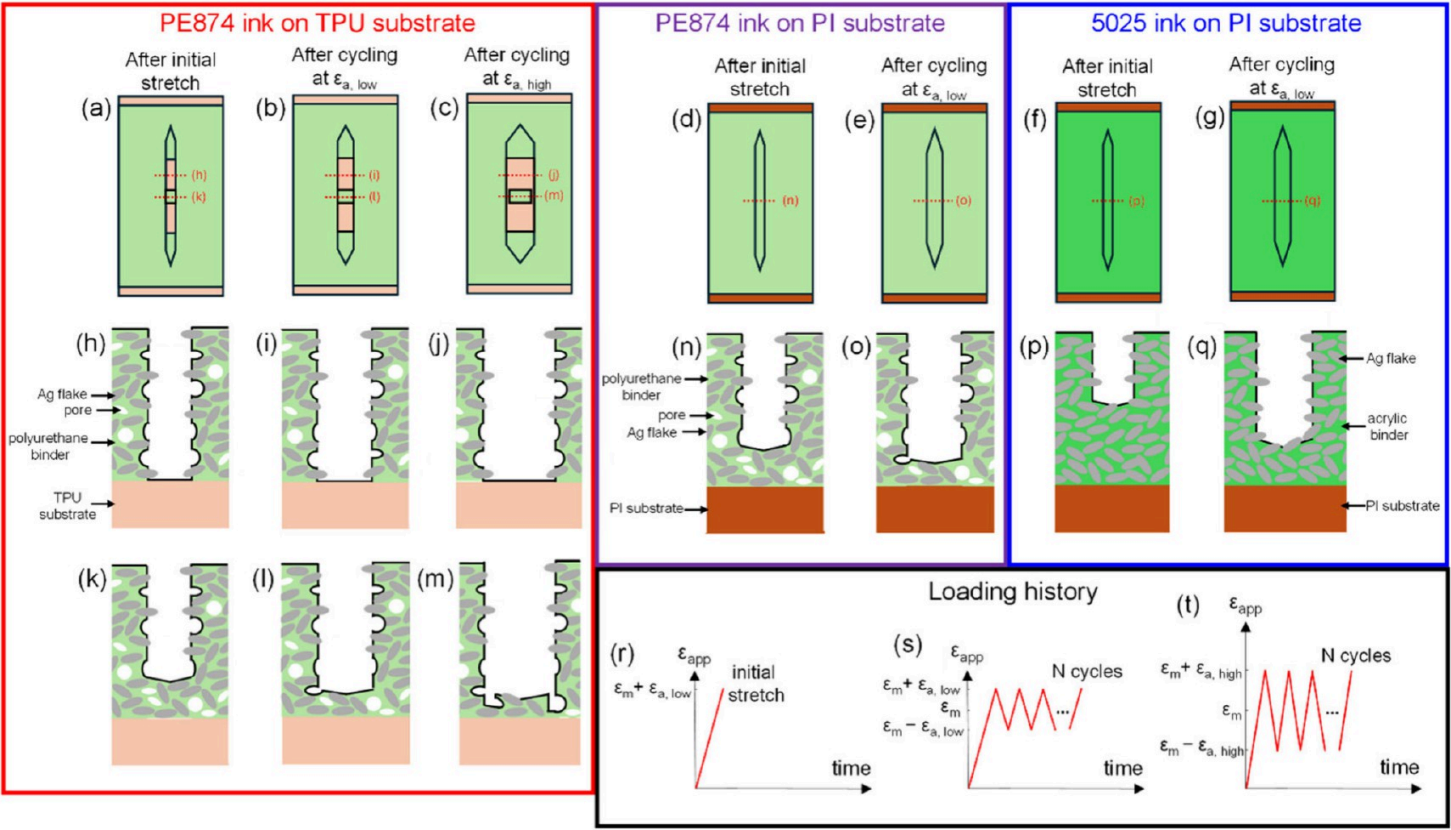
Plan view schematic of cracks after monotonic stretch for (a) PE874-TPU, (d) PE874-PI, and (g) 5025-PI, after subsequent cyclic stretching at low strain amplitude for (b) PE874-TPU, (e) PE874-PI, and (g) 5025-PI, and (c) after subsequent cyclic stretching at high strain amplitude for PE874-TPU; (h–q) corresponding cross-sectional schematics for cross sections labeled by red dotted lines in (a–g) (different length scale than (h–q)); loading history schematics for (r) initial monotonic stretch, (s) cycling at low strain amplitude ε_a, low_, and (t) cycling at high strain amplitude ε_a, high_.

Figure 9 shows the plan (Figure 9(a–g)) and cross-sectional (Figure 9(h–q)) view schematics of representative cracks summarizing the cracking mechanisms for different material properties and strain amplitudes. The plan and cross-sectional view schematics show a representative crack in the ink layer for the three discussed ink–substrate systems after the initial monotonic stretching and after subsequent cyclic stretching. After the initial monotonic stretch, the crack in PE874 on TPU substrate is through-thickness along the greater part of its length, but there is still some bridging ink material near the crack tip as well as in the crack wake (Figure 9(a)). After cyclic straining at low strain amplitude, the crack width is increased (Figure 9(b)). Figure 9(h,i),(k,l) shows the widening of the crack in the through-thickness and partially through-thickness crack cross sections, respectively. Though a large portion of the crack is through-thickness, the fatigue damage by the crack widening and penetration through the still intact bridging ink material is responsible for the ink’s electrical degradation. Figure 9(f,g),(p,q) shows, respectively, the plan and cross-section view of the crack widening for the 5025 ink on PI substrate cycled at low strain amplitude. Unlike the PE874 ink on the TPU substrate, the entire crack in the 5025 ink on the PI substrate is only partially through the ink thickness after the initial stretch (Figure 9(f)). After cyclic straining, the bridging material along the entire length of the crack in the 5025 ink on PI substrate is damaged by the crack widening and penetration into the ink thickness.

The substrate material also has a significant effect on the cracking behavior. The PI substrate has a much higher elastic modulus than the TPU substrate (∼3 GPa for PI compared to ∼10 MPa for TPU) and provides more constraint for strain localization in the ink layer.^43^ Previous in situ SEM monotonic stretch experiments showed that the PI substrate constrained the cracks and prevented the cracks from penetrating the ink thickness not only in the 5025 ink but also in the PE874 ink when the PE874 ink is printed on the PI substrate (Supporting Information Figure S8, S9, and S10), resulting in a denser, more diffuse crack pattern (Supporting Information Figure S12) with more partial through-thickness cracks. The cracks in the PE874 ink on the PI substrate have more conductive bridging ink material compared to the PE874 ink on the TPU substrate, where through-thickness cracking was prevalent. The measured *R*/*R*_0_ at maximum strain during the initial cycle was slightly lower (4.4 compared to 5.6 for PE874-TPU; see Supporting Information Figure S11) due to less through-thickness cracking. Upon cycling, however, (*R*/*R*_0_)_min_^′^ for the PE874-PI was significantly higher (Figure 3) due to the larger amount of bridging ink material being susceptible to more fatigue damage. It should also be noted that, compared with the 5025 ink on the PI substrate, the PE874 ink on the PI substrate still has less bridging ink material due to the deeper crack penetration in the PE874 ink. This difference could explain the higher (*R*/*R*_0_)_min_^′^ for the 5025-PI compared to the PE874-PI (Figure 3). Figure 9(d,e),(n,o) shows the widening of a representative crack in the PE874 ink on the PI substrate. Unlike on the TPU substrate, the crack in the PE874 ink on PI substrate is only partially through the ink thickness (Figure 9(d)), but it has less bridging material compared to the 5025 ink on PI substrate (Figure; 9(p)). With cyclic straining, the PE874 ink on the PI substrate is more prone to fatigue damage compared to the same ink on the TPU substrate.

### 3.6 Discussion: Effect of Strain Amplitude on Cracking Behavior

The strain amplitude is known to have a strong correlation with the rate of *R*/*R*_0_ increase with cycling. The DIC relative strain maps in Figures 3 and 4 show that the magnitude of relative strains Δε_*xx*_ and Δε_*xy*_ for the cracks in the 10 ± 5% and 40 ± 15% in situ experiments with PE874-TPU showed a direct correlation with the magnitude of (*R*/*R*_0_)′ of the two respective experiments during the first 5 cycles. Therefore, larger crack widening and shear displacements correspond to a larger increase in *R*/*R*_0_ per cycle. The cracking mechanism responsible for this correlation is that the larger local strains from crack widening and shearing cause relatively larger separation of the intact bridging material (mainly Ag flakes) per cycle, leading to a higher *R*/*R*_0_ increase. Figure 9(j,m) shows the corresponding through-thickness and partial through-thickness cross sections after cyclic stretching at a higher strain amplitude, in which case there is larger crack widening resulting in more damage to the bridging ink material compared to the case in Figure 9(i,l).

## 4 Conclusions

From the in situ SEM cyclic stretch tests performed on the PE874 ink on the TPU substrate and 5025 ink on the PI substrate, the following conclusions are drawn about the relation between strain localization and electrical resistance increase with cyclic straining for this class of conductive ink materials.

The increase in ink electrical resistance with uniaxial cyclic strain can be directly related to the progressive fatigue damage in the cracked regions by mixed mode crack widening and shearing displacements over the cycles. For the PE874 ink, there appears to be a correlation between the rate of change in resistance with cycling and the relative increase in axial and shear local strain over the cycles. Higher applied strain amplitudes generate higher relative increases in axial and shear strains.

Cyclic strain does not significantly increase the extent of the crack pattern established during the initial monotonic stretching of the first cycle. The increase in ink resistance with cycling comes from the fatigue damage to the intact ink material within the existing crack pattern by the widening and shearing of the cracks. All of the ink cracking is limited to in-plane cracks and penetration of cracks through the ink thickness, with no delamination occurring between the ink and substrate layers.

The ink material properties are in large part responsible for the different cracking mechanisms, and therefore, the rates of *R*/*R*_0_ increase in the two inks. The PE874 ink is associated with through-thickness cracking, whereas the 5025 ink material is more resistant to through-thickness cracking and only has partial through-thickness cracks with more conductive bridging ink material. Under cyclic stretching, the 5025 ink is susceptible to more fatigue damage to the larger amount of bridging ink material being damaged, leading to larger increases in *R*/*R*_0_.

The substrate can also play a secondary role in the ink cracking behavior, and resistance increases with cyclic strain. A stiff substrate constrains strain localization and reduces the extent of crack penetration through the ink thickness. The cracks in the PE874 ink on the PI substrate have more conductive bridging ink material than those in the PE874 ink on the TPU substrate. Consequently, the PE874 ink on the PI substrate has a higher rate of resistance increase with cyclic strain than the PE874 ink on the TPU substrate.

The detailed knowledge of cracking mechanisms during cyclic stretch gained from the in situ SEM experiments with the two inks has allowed for a more extensive understanding of how the material properties, fatigue damage mechanisms, and electrical performance are interrelated for this class of conductive ink materials. The insight gained from these experiments should prove valuable for the future design of these conductive inks with the goal of minimizing the electrical resistance increase during cyclic deformation.

## Supporting Information Available

Figure S1: Axial strain maps with images at 0% applied strain as reference image for PE874-TPU 10 ± 5% at cycle 1, PE874-TPU 10 ± 5% at cycle 20, 5025-PI 10 ± 5% at cycle 1, and 5025-PI 10 ± 5% at cycle 20. Figure S2: PE874-TPU in situ SEM fatigue test at 10 ± 5% strain: 500× images at maximum strain during cycle 1 and cycle 20; relative tensile strain Δε_*xx*_ maps for cycle 2, cycle 5, cycle 10, and cycle 20; relative shear strain Δε_*xy*_ maps for cycle 2, cycle 5, cycle 10, andcycle 20 (same length scale as the 500× images); relative strain plotted over marked paths on DIC maps for Δε_*xx*_ and Δε_*xy*_; relative strain peaks for selected cracks for Δε_*xx*_ and Δε_*xy*_. Figure S3: PE874-TPU in situ SEM fatigue test at 40 ± 15% strain: 500× images at maximum strain during cycle 1 and cycle 20; relative tensile strain Δε_*xx*_ maps for cycle 2, cycle 5, cycle 10, and cycle 20; relative shear strain Δε_*xy*_ maps for cycle 2, cycle 5, cycle 10, and cycle 20 (same length scale as the 500× images); relative strain plotted over marked paths on DIC maps for Δε_*xx*_ and Δε_*xy*_; relative strain peaks for selected cracks for Δε_*xx*_ and Δε_*xy*_. Figure S4: 5025-PI in situ SEM fatigue test at 10 ± 5% strain: 500× images at maximum strain during cycle 1 and cycle 20; relative tensile strain Δε_*xx*_ maps for cycle 2, cycle 5, cycle 10, and cycle 20; relative shear strain Δε_*xy*_ maps for cycle 2, cycle 5, cycle 10, and cycle 20 (same length scale as the 500× images); relative strain plotted over marked paths on DIC maps for Δε_*xx*_ and Δε_*xy*_; relative strain peaks for selected cracks for Δε_*xx*_ and Δε_*xy*_. Figure S5: Close-up images and corresponding relative strain maps (same length scale) over cycles for a representative widening crack from PE874-TPU test at 10 ± 5% strain. Figure S6: Close-up images and corresponding relative strain maps (same length scale) over cycles for a representative widening crack from PE874-TPU test at 40 ± 15% strain. Figure S7: Close-up images and corresponding relative strain maps (same length scale) over cycles for a representative widening crack from the 5025-PI test at 10 ± 5% strain. Figure S8: Evolution of a crack in PE874 ink on PI substrate over applied strains. Figure S9: Evolution of a crack in PE874 ink on TPU substrate over applied strains. Figure S10: Evolution of a crack in 5025 ink on PI substrate over applied strains. Figure S11: *R*/*R*_0_ evolution with applied strain for PE874-TPU and PE874-PI. Figure S12: Crack pattern comparison between PE874-TPU, PE874-PI, and 5025-PI based on DIC strain maps according to the 2ε_app_ criterion (PDF)
