# Supplementary material for: In Situ Scanning Electron Microscopy Crack Characterization and Resistance Evolution in Cyclically-Strained Ag Nanoflake-Based Inks
Source: ACS Appl Nano Mater. 2024 Nov 25;7(23):27173–84. doi: 10.1021/acsanm.4c05133 (PMC11650634; doi:10.1021/acsanm.4c05133)

SUPPORTING INFORMATION

In Situ Scanning Electron Microscopy Crack Characterization and Resistance Evolution in Cyclically-Strained Ag Nanoflake-Based Inks

Qiushi Li, Antonia Antoniou* and Olivier Pierron*

G.W. Woodruff School of Mechanical Engineering, Georgia Institute of Technology, Atlanta, GA, 30318, USA

Emails: antonia.antoniou@me.gatech.edu, olivier.pierron@me.gatech.edu

Figure S1

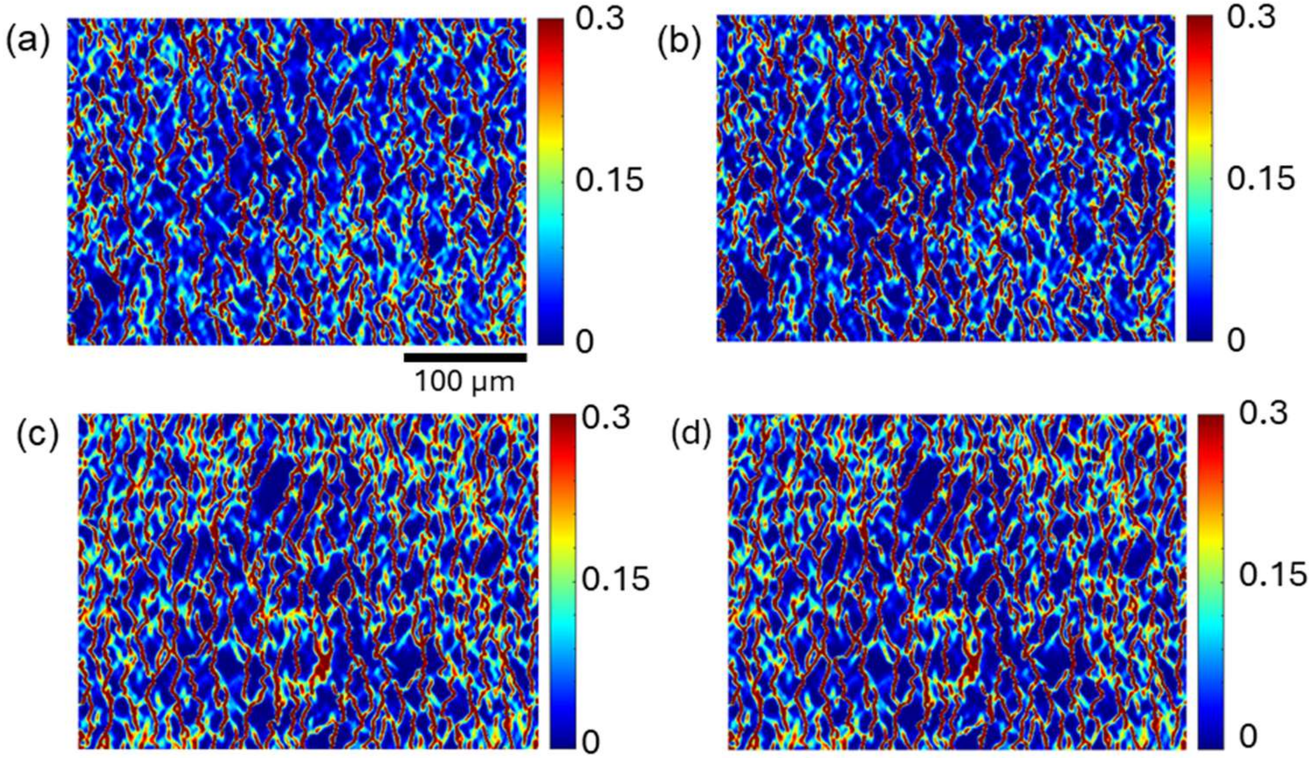

Axial strain maps with images at 0% applied strain as reference image for (a) PE874-TPU 10±5% at cycle 1, (b) PE874-TPU 10±5% at cycle 20, (c) 5025-PI 10±5% at cycle 1, (d) 5025-PI 10±5% at cycle 20.

Figure S2

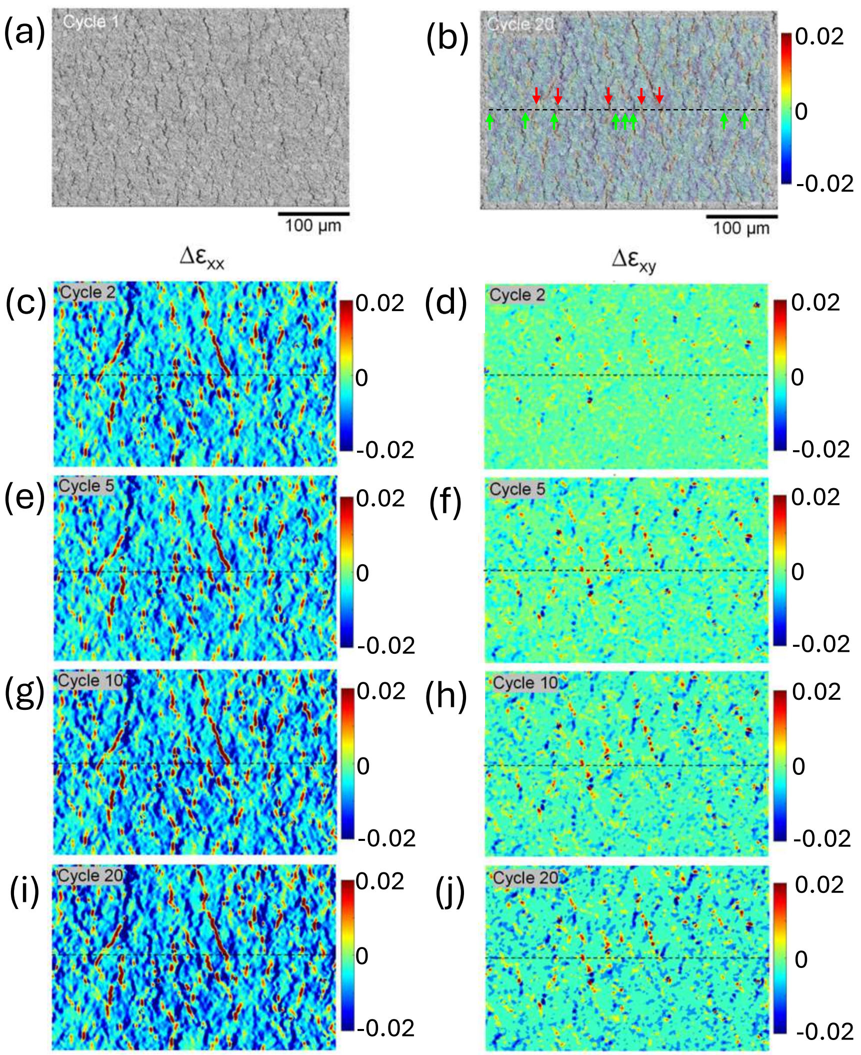

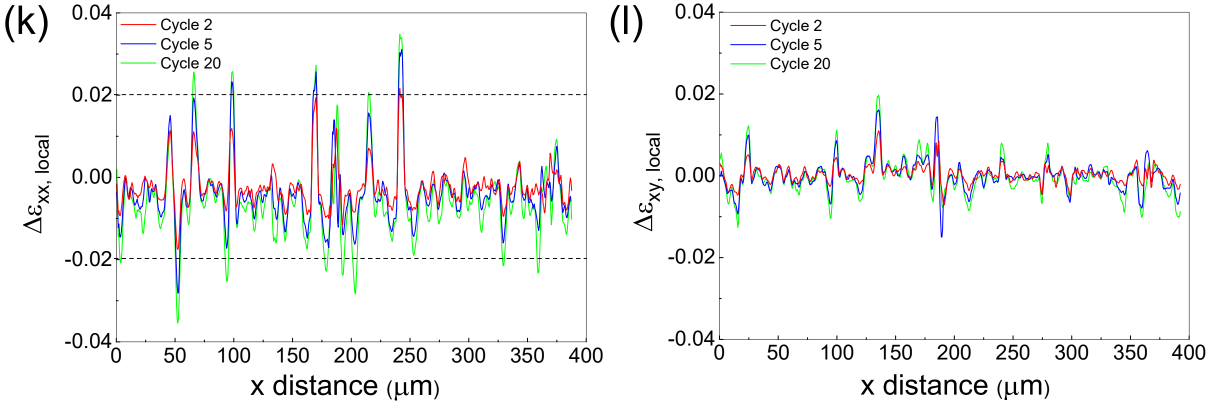

For PE874-TPU in-situ SEM fatigue test at 10±5% strain: 500x images at maximum strain during (a) cycle 1 and (b) cycle 20; relative tensile strain $\Delta\varepsilon_{xx}$ maps for (c) cycle 2, (e) cycle 5, (g) cycle 10, and (i) cycle 20; relative shear strain $\Delta\varepsilon_{xy}$ maps for (d) cycle 2, (f) cycle 5, (h) cycle 10, and (j) cycle 20 (same length scale as the 500x images); relative strain plotted over marked paths on DIC maps for (k) $\Delta\varepsilon_{xx}$ and (l) $\Delta\varepsilon_{xy}$; relative strain peaks for selected cracks for (m) $\Delta\varepsilon_{xx}$ and (n) $\Delta\varepsilon_{xy}$.

Widening cracks are marked with red arrows and narrowing cracks are marked with green arrows on figure part (b), which has the $\Delta\varepsilon_{xx}$ relative axial strain map superposed on the SEM image. Ratio of widening to narrowing cracks: 5:8

Figure S3

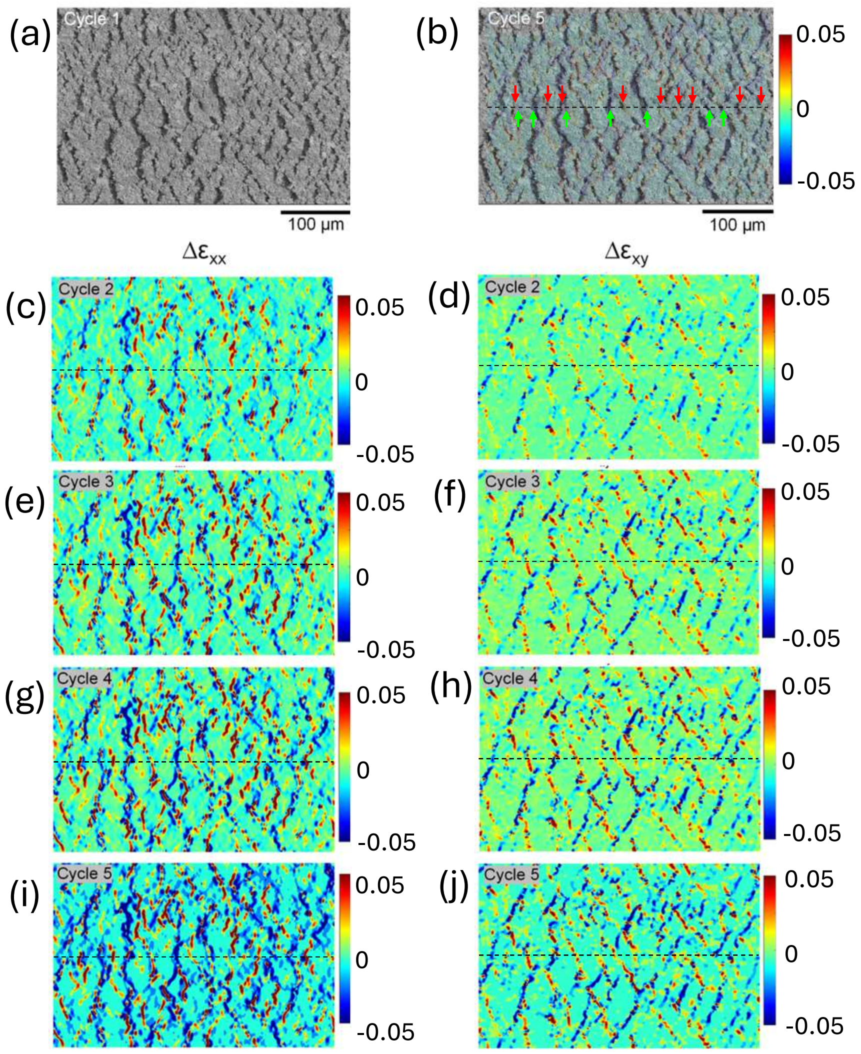

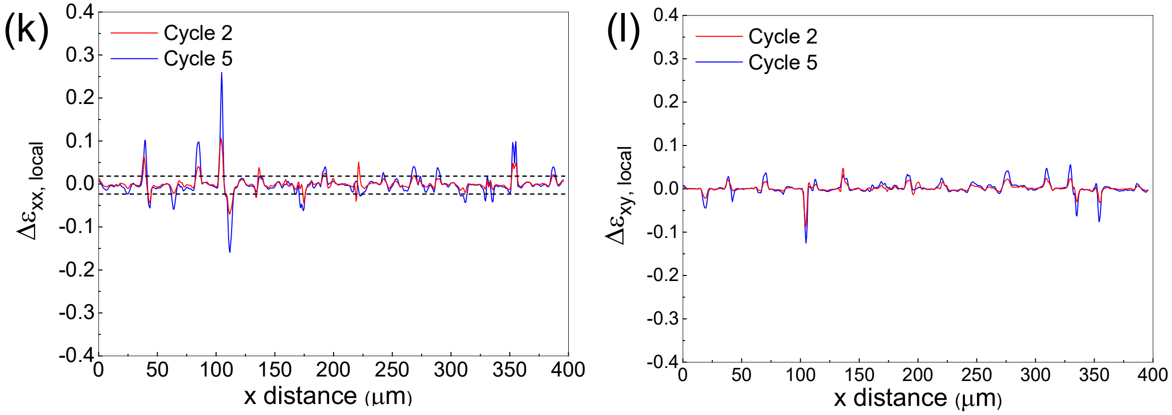

For PE874-TPU in-situ SEM fatigue test at 40±15% strain: 500x images at maximum strain during (a) cycle 1 and (b) cycle 20; relative tensile strain $\Delta\varepsilon_{xx}$ maps for (c) cycle 2, (e) cycle 5, (g) cycle 10, and (i) cycle 20; relative shear strain $\Delta\varepsilon_{xy}$ maps for (d) cycle 2, (f) cycle 5, (h) cycle 10, and (j) cycle 20 (same length scale as the 500x images); relative strain plotted over marked paths on DIC maps for (k) $\Delta\varepsilon_{xx}$ and (l) $\Delta\varepsilon_{xy}$; relative strain peaks for selected cracks for (m) $\Delta\varepsilon_{xx}$ and (n) $\Delta\varepsilon_{xy}$.

Widening cracks are marked with red arrows and narrowing cracks are marked with green arrows on figure part (b), which has the $\Delta\varepsilon_{xx}$ relative axial strain map superposed on the SEM image. Ratio of widening to narrowing cracks: 9:7

Figure S4

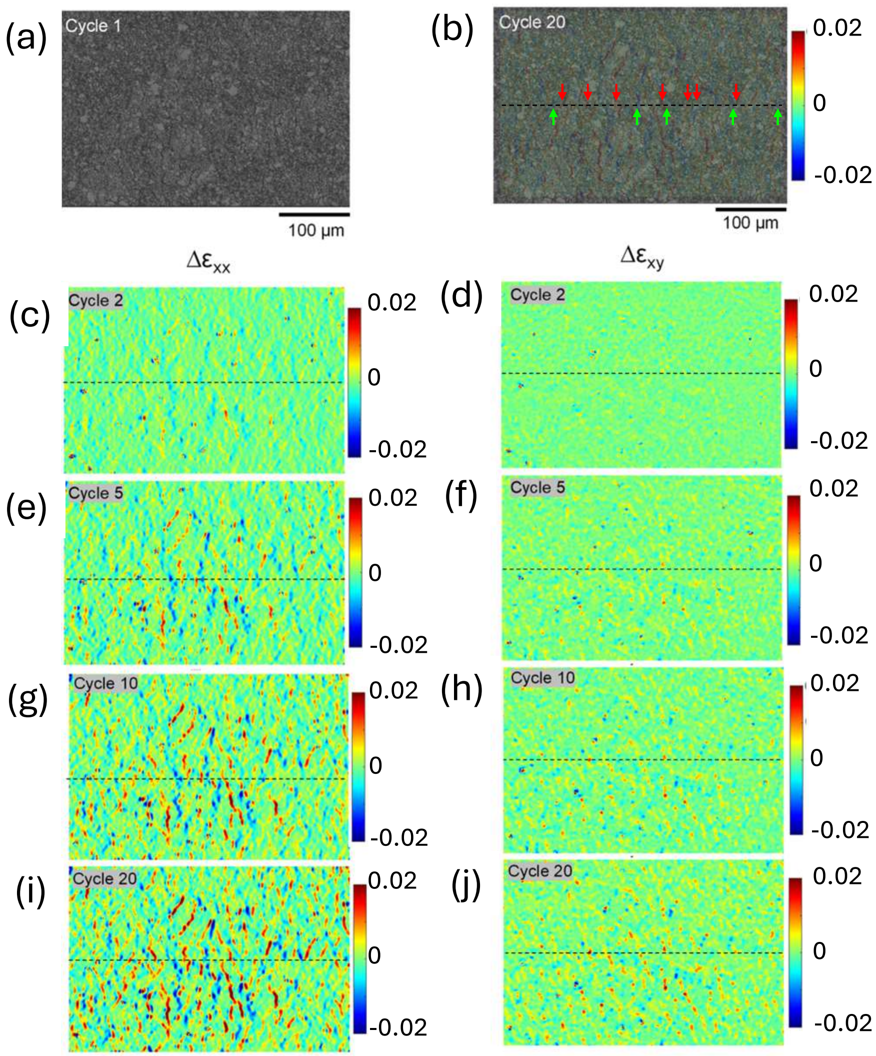

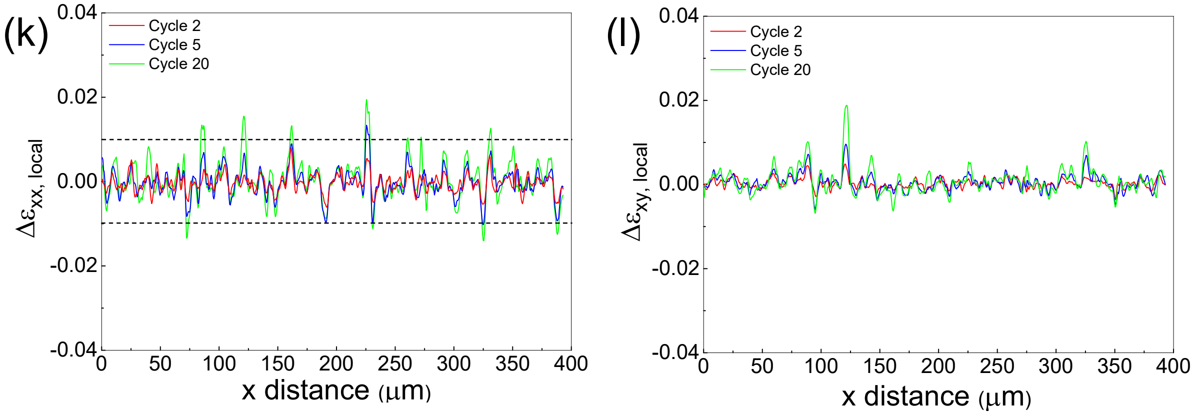

For 5025-PI in-situ SEM fatigue test at 10±5% strain: 500x images at maximum strain during (a) cycle 1 and (b) cycle 20; relative tensile strain $\Delta\varepsilon_{xx}$ maps for (c) cycle 2, (e) cycle 5, (g) cycle 10, and (i) cycle 20; relative shear strain $\Delta\varepsilon_{xy}$ maps for (d) cycle 2, (f) cycle 5, (h) cycle 10, and (j) cycle 20 (same length scale as the 500x images); relative strain plotted over marked paths on DIC maps for (k) $\Delta\varepsilon_{xx}$ and (l) $\Delta\varepsilon_{xy}$; relative strain peaks for selected cracks for (m) $\Delta\varepsilon_{xx}$ and (n) $\Delta\varepsilon_{xy}$.

Widening cracks are marked with red arrows and narrowing cracks are marked with green arrows on figure part (b), which has the $\Delta\varepsilon_{xx}$ relative axial strain map superposed on the SEM image. Ratio of widening to narrowing cracks: 7:5

Figure S5 - Closeup images and corresponding relative strain maps (same length scale) over cycles for a representative widening crack from PE874-TPU test at 10±5% strain.

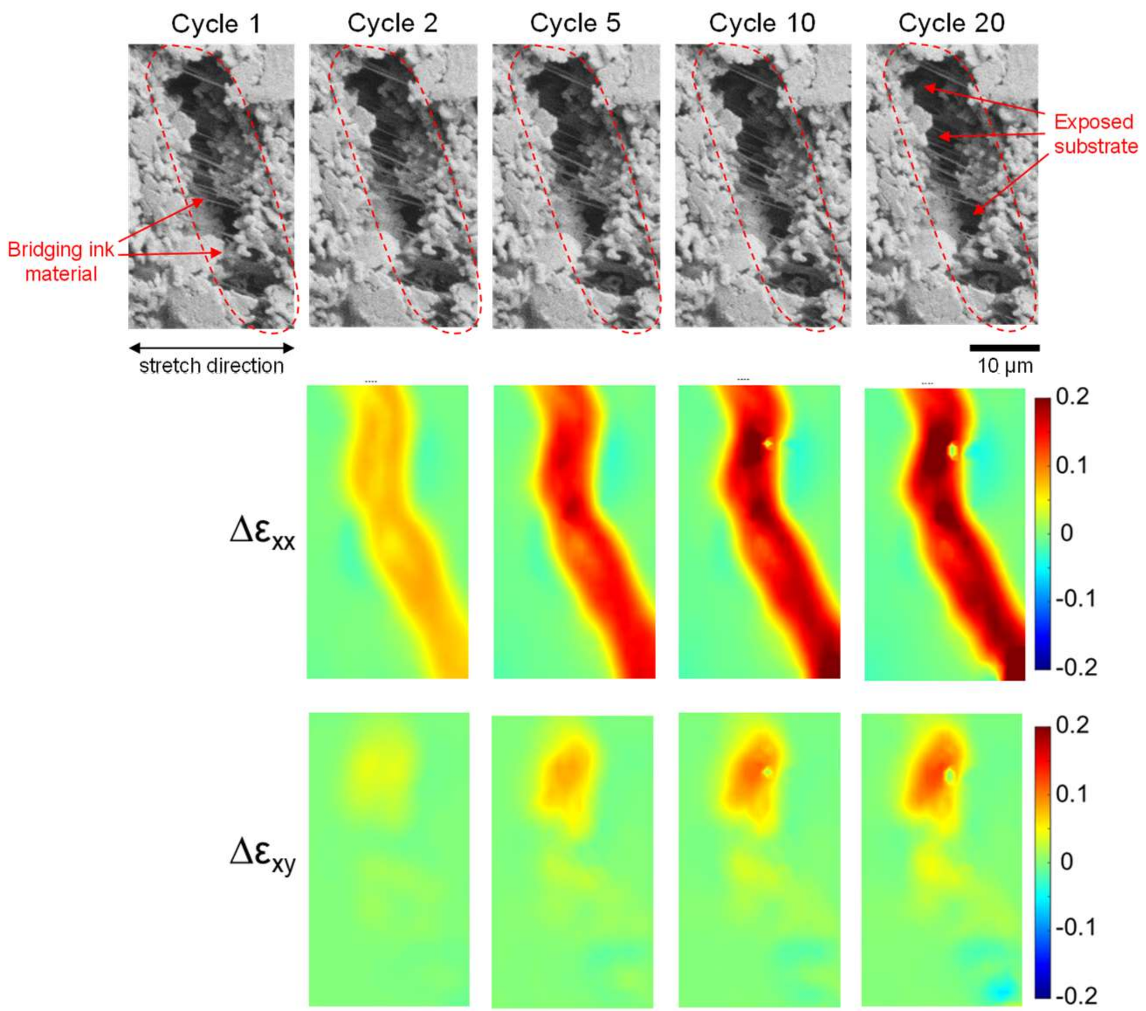

Figure S6 – Closeup images and corresponding relative strain maps (same length scale) over cycles for a representative widening crack from PE874-TPU test at 40±15% strain.

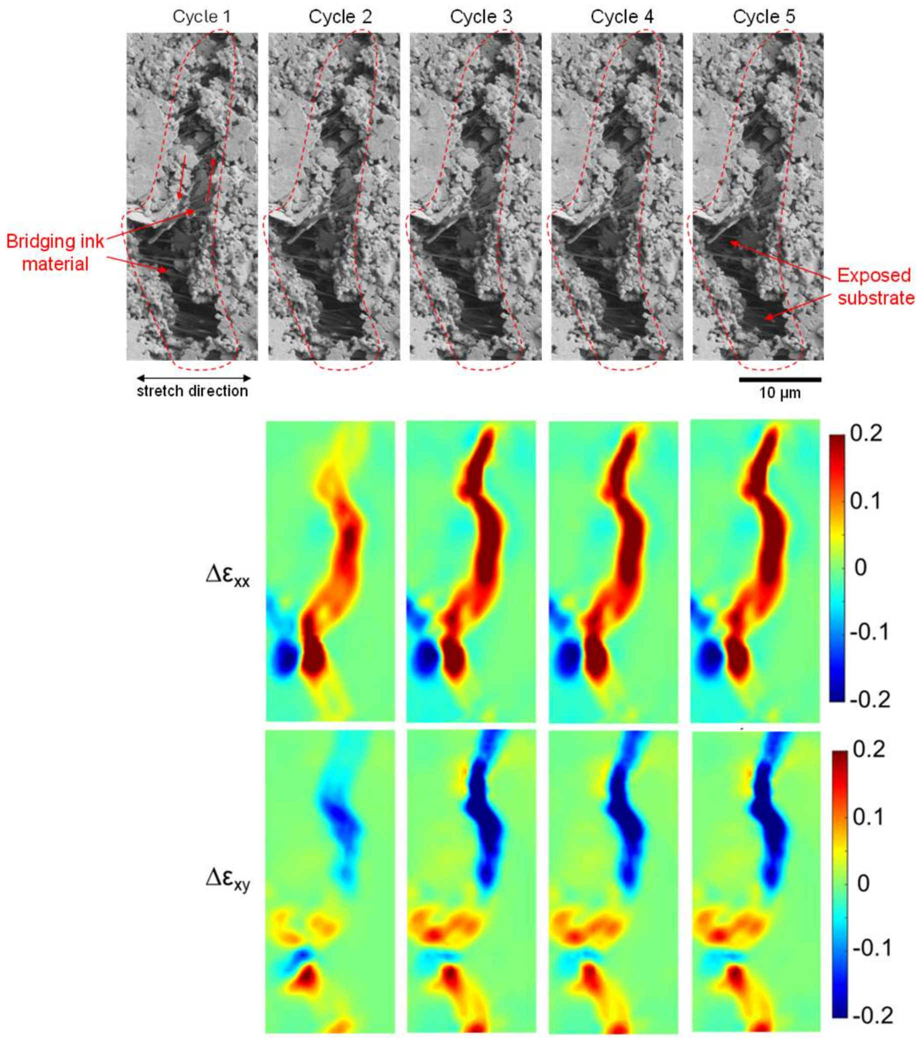

Figure S7 – Closeup images and corresponding relative strain maps (same length scale) over cycles for a representative widening crack from 5025-PI test at 10±5% strain.

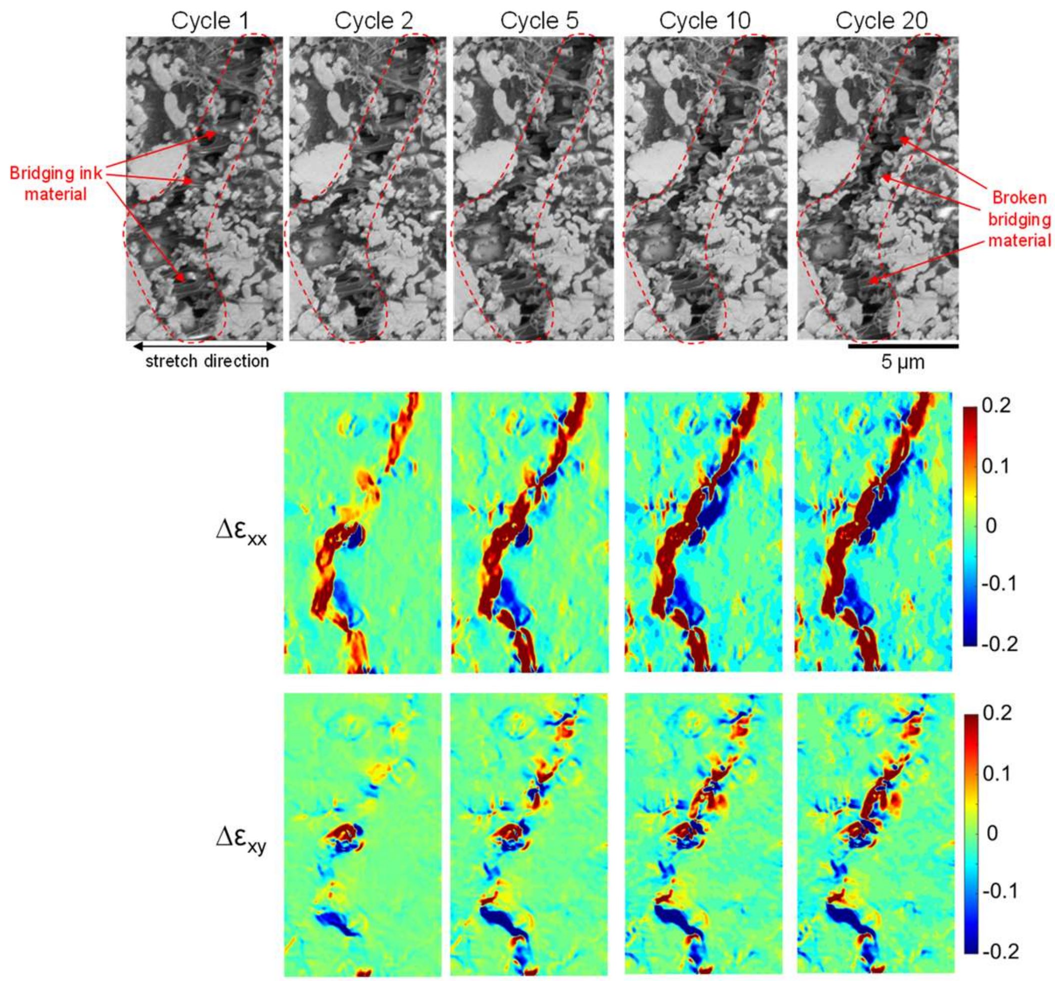

Figure S8 – Evolution of a crack in PE874 ink on PI substrate over applied strains

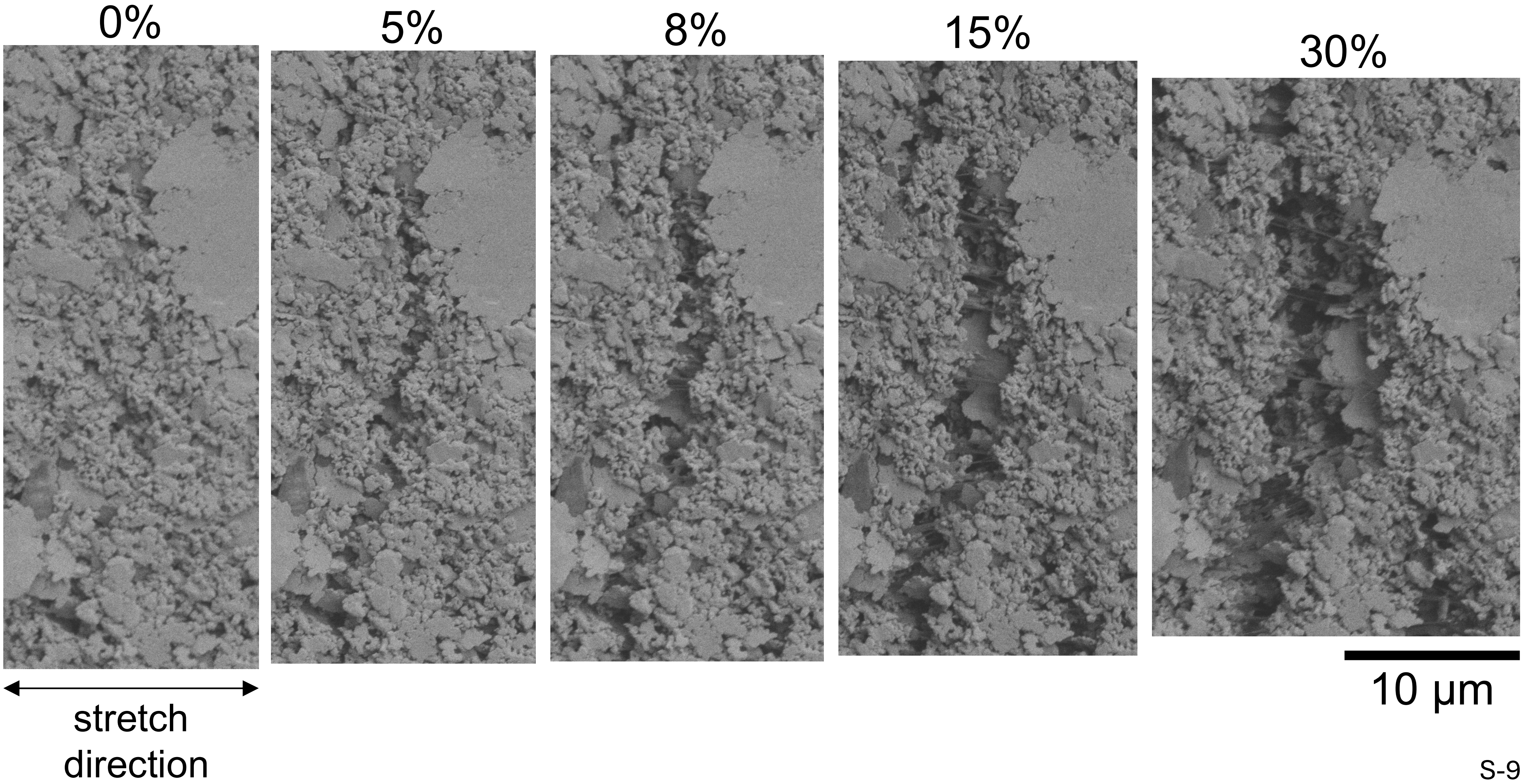

Figure S9 – Evolution of a crack in PE874 ink on TPU substrate over applied strains

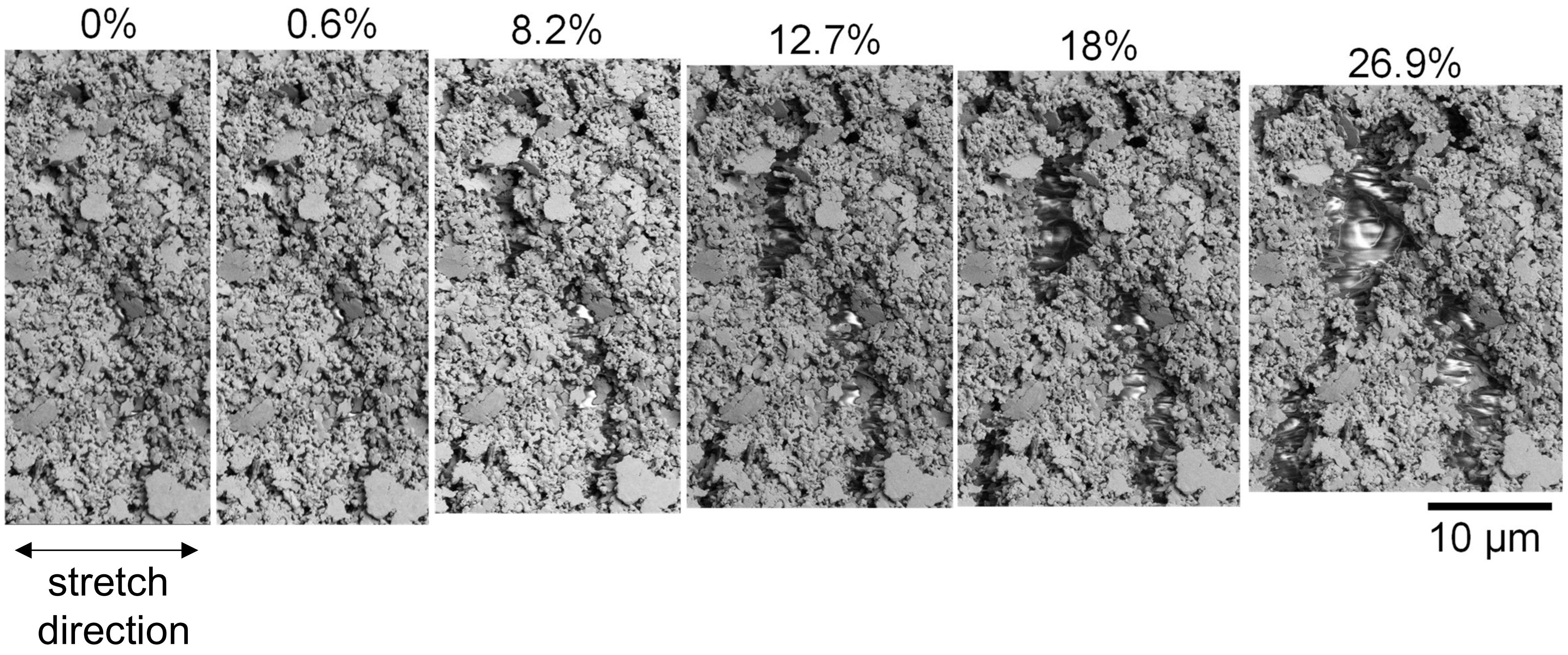

Figure S10 – Evolution of a crack in 5025 ink on PI substrate over applied strains

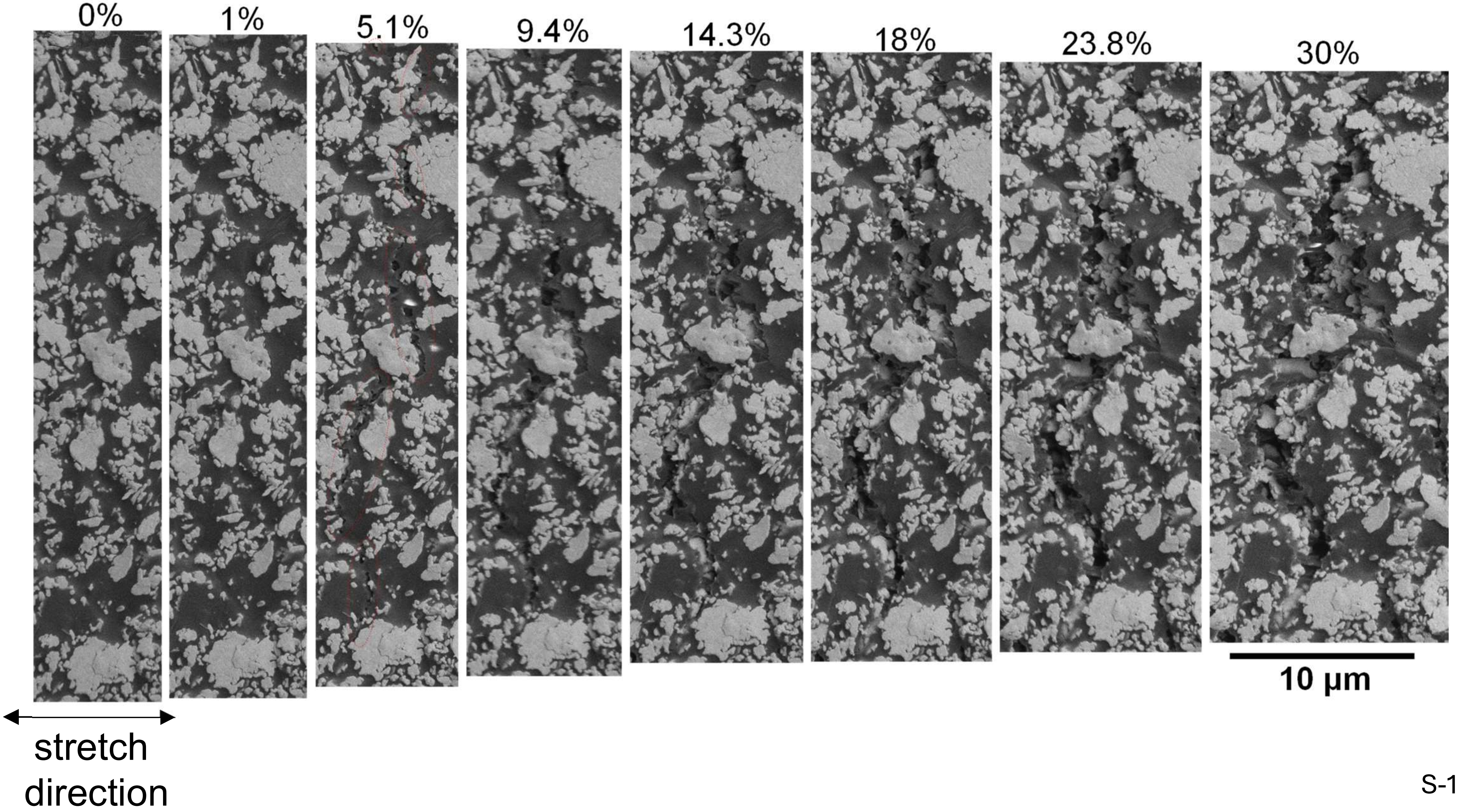

Figure S11 – R/R_0 evolution with applied strain for PE874-TPU and PE874-PI

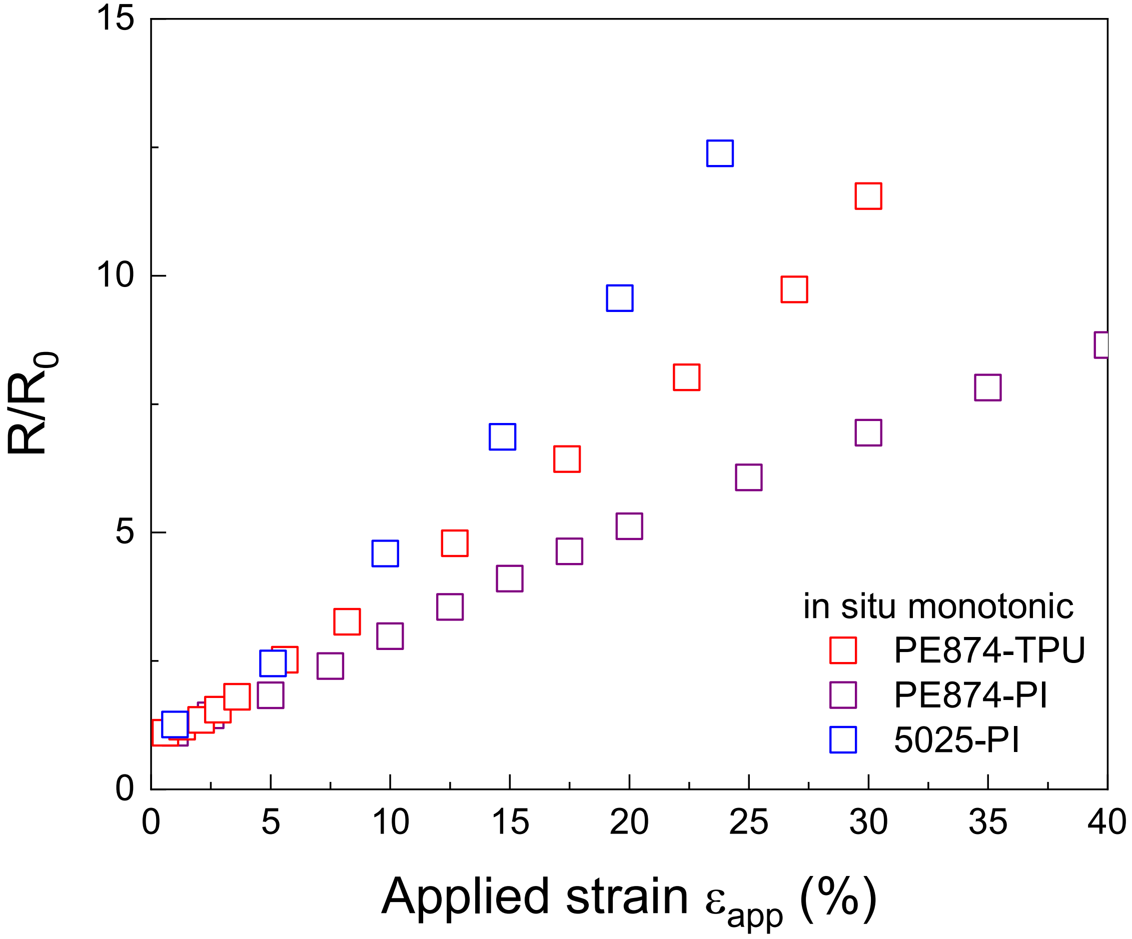

Figure S12 – Crack pattern comparison between PE874-TPU, PE874-PI, and 5025-PI based on DIC strain maps according to the $2\varepsilon_{app}$ criterion

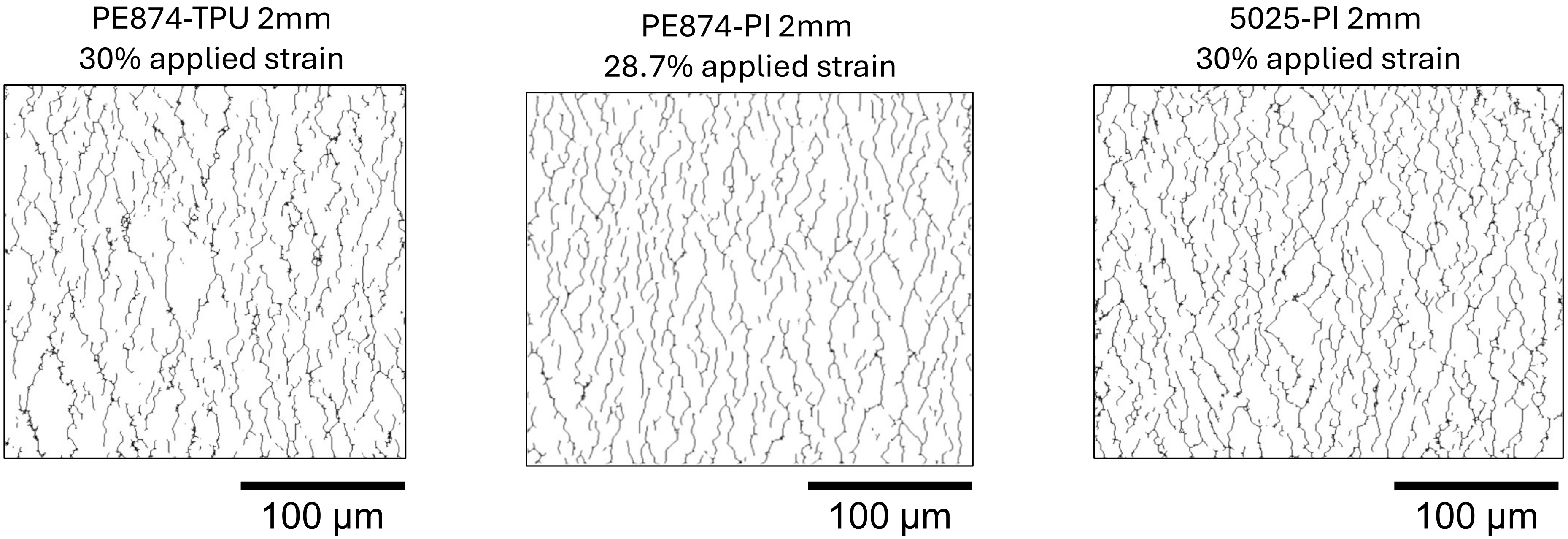

Supplement: Supplementary file 1 — an4c05133_si_001.pdf [file an4c05133_si_001.pdf]
